# True Nature of the Transition-Metal Carbide/Liquid Interface Determines Its Reactivity

**DOI:** 10.1021/acscatal.1c00415

**Published:** 2021-04-07

**Authors:** Christoph Griesser, Haobo Li, Eva-Maria Wernig, Daniel Winkler, Niusha Shakibi Nia, Thomas Mairegger, Thomas Götsch, Thomas Schachinger, Andreas Steiger-Thirsfeld, Simon Penner, Dominik Wielend, David Egger, Christoph Scheurer, Karsten Reuter, Julia Kunze-Liebhäuser

**Affiliations:** † Department of Physical Chemistry, University of Innsbruck, Innrain 52c, 6020 Innsbruck, Austria; ‡ Chair of Theoretical Chemistry and Catalysis Research Center, Technische Universität München, 85748 Garching, Germany; § Department of Heterogeneous Reactions, Max Planck Institute for Chemical Energy Conversion, Stiftstraße 34-36, 45470 Mülheim an der Ruhr, Germany; ∥ Department of Inorganic Chemistry, Fritz-Haber-Institut der Max-Planck-Gesellschaft, Faradayweg 4-6, 14195 Berlin, Germany; ⊥ University Service Center for Transmission Electron Microscopy, TU Wien, 1040 Vienna, Austria; # Linz Institute for Organic Solar Cells (LIOS)/Institute of Physical Chemistry, Johannes Kepler University, 4040 Linz, Austria; ∇ Fritz-Haber-Institut der Max-Planck-Gesellschaft, Faradayweg 4-6, 14195 Berlin, Germany

**Keywords:** electrocatalysis, transition-metal carbides, electrochemical CO_2_ reduction, surface Pourbaix diagram, ab initio thermodynamics, solid/liquid interface, XPS, HER

## Abstract

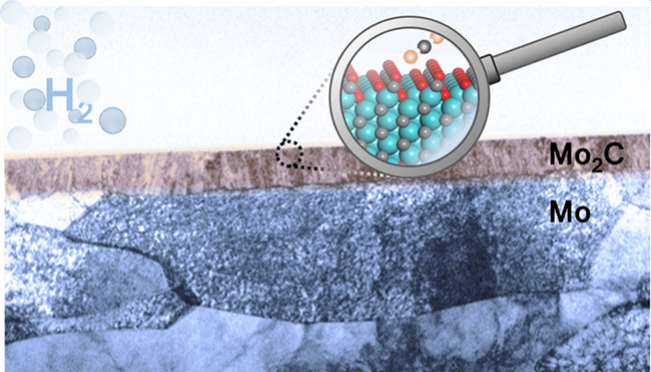

Compound materials, such as transition-metal (TM) carbides, are anticipated to be effective electrocatalysts for the carbon dioxide reduction reaction (CO_2_RR) to useful chemicals. This expectation is nurtured by density functional theory (DFT) predictions of a break of key adsorption energy scaling relations that limit CO_2_RR at parent TMs. Here, we evaluate these prospects for hexagonal Mo_2_C in aqueous electrolytes in a multimethod experiment and theory approach. We find that surface oxide formation completely suppresses the CO_2_ activation. The oxides are stable down to potentials as low as −1.9 V versus the standard hydrogen electrode, and solely the hydrogen evolution reaction (HER) is found to be active. This generally points to the absolute imperative of recognizing the true interface establishing under operando conditions in computational screening of catalyst materials. When protected from ambient air and used in nonaqueous electrolyte, Mo_2_C indeed shows CO_2_RR activity.

## 1 Introduction

Transition-metal compounds are widely applicable as catalyst or catalyst support materials in heterogeneous catalysis. The reason for this is their structural and compositional diversity, which can be adopted to the reaction of interest and which can suppress the correlation of adsorption energies, a phenomenon frequently referred to as scaling relations.^1^ Such scaling is made responsible for numerous limitations in heterogeneous catalysis on transition metals (TMs),^2,3^ but can be overcome by compound materials, such as oxides,^4^ sulfides, or carbides.^2,3,5−7^ High prospects for usage in thermal catalysis^8−19^ and electrocatalysis^6^ are therefore assigned to TM carbides (TMCs) as least explored materials class among these compound catalysts. Their ability to uptake and activate CO_2_ and thus drive the eminent electrochemical carbon dioxide reduction reaction (CO_2_RR) has recently been predicted by a number of theoretical works.^20−22^ The two most severe generic issues in the CO_2_RR are the competing hydrogen evolution reaction (HER) that often dominates in aqueous electrolytes^6^ and the adsorption energy scaling of CO and CHO, which are central reaction intermediates.^23^ And indeed, for a range of single-crystal TMC surfaces, the density functional theory (DFT) calculations, underlying those promising forecasts, indicated a breaking of this key limiting scaling^23^ due to a more carbophobic and oxophilic nature in comparison to the parent metals.^7,20,21^ Such guiding predictions often made in the context of larger computational screening studies^2,3^ are essential for experimentalists because the chemical diversity of compound materials is too vast for empirical approximation.

In particular for Mo_2_C, an active-site computational screening study performed by some of us investigated the numerous and various active sites offered by this TMC and further confirmed its high suitability for the CO_2_RR.^7^ Irritatingly, despite all this praise, only one experimental study of the CO_2_RR on Mo_2_C powder and sheets has been reported to date.^24^ A minuscule conversion to methane (CH_4_) with a Faradaic efficiency of <0.1% was detected at ≤−0.95 V versus the standard hydrogen electrode (SHE), denoted as *V*_SHE_ in the following, while the dominant current was related to the competing hydrogen evolution reaction (HER).

Aiming to understand this blatant discrepancy between claim and reality, we here perform a multimethod approach to investigate Mo_2_C’s CO_2_ electroreduction capability and the related surface chemistry in aqueous electrolyte. Our experiments confirm an in fact exclusive activity toward the HER, without any formation of CO_2_ reduction products. In the search for an explanation, we realize that the bulk material and especially the parent metal are prone to oxidation, even at rather negative potentials. Detailed ab initio thermodynamics calculations reveal that the situation at the carbide surface is even worse than expected from the generic bulk Pourbaix (*E*/pH) diagrams of Mo_2_C^25^ and its parent metal.^26,27^ In the relevant potential range, the carbide surface is always covered by oxygenated species, and it is the HER activity of these oxidic films that is actually seen in the experiments. This understanding is fully confirmed by an experimental surface *E*/pH diagram recorded with quasi *in-situ* X-ray photoelectron spectroscopy (XPS)^28^ under consideration of surface pH effects. To one end, these findings underscore the urgent need to extend computational screening studies from their present evaluation of the bulklike material to an account of the catalyst surface structure and composition that is actually forming operando.^29,30^ On the other hand, given the highly appealing properties of the carbide motivates us to search for experimental reaction conditions where these properties may indeed be harvested. First experiments correspondingly performed in nonaqueous electrolyte immediately show a high CO_2_ reduction activity of the then nonoxidized or barely oxidized Mo_2_C.

## 2 Results and Discussion

### 2.1 Electrocatalytic Performance of Planar Mo_2_C Films

**Figure 1:**
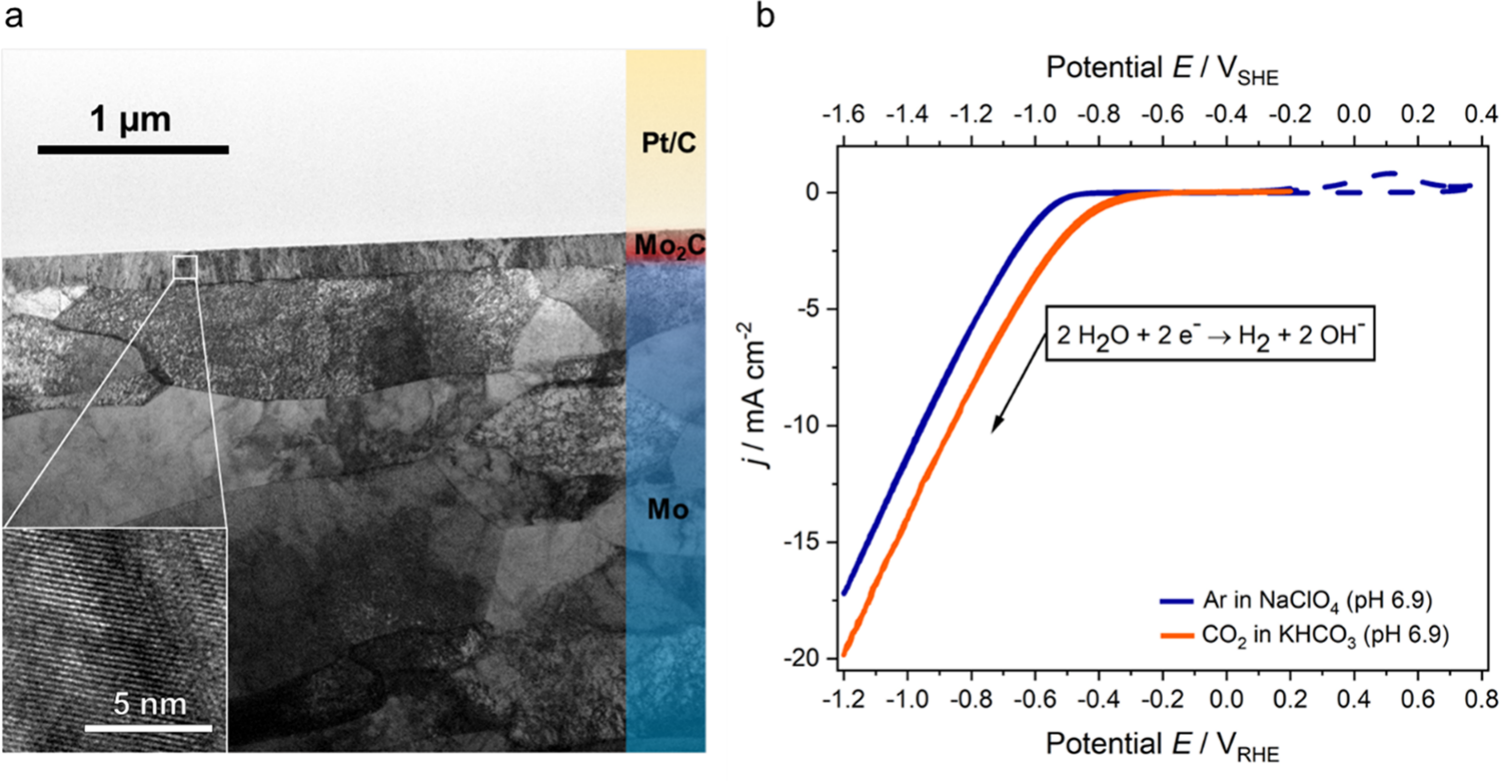
Mo_2_C film cross section and structure, and its cathodic water reduction activity. (a) Bright-field (BF) TEM image of the Mo_2_C film in a focused ion beam (FIB) lamella embedded between the polycrystalline Mo substrate and a Pt/C protection layer, and HRTEM image of the Mo_2_C film (inset). (b) Cathodic scans of Mo_2_C recorded at 20 mV s^–1^ in 0.1 M CO_2_-saturated KHCO_3_ (orange line) and Ar-saturated 0.1 M NaClO_4_ (blue line), both at pH 6.9. The dashed blue line shows an independently recorded cyclic voltammogram (CV) featuring anodic oxidation (see text); *j* is the current density, and *E* is the electrode potential versus the standard hydrogen electrode (SHE, upper axis) or the reversible hydrogen electrode (RHE, lower axis).

The planar molybdenum carbide (Mo_2_C) films that we synthesized for our study consist of large, several-micrometer-wide terraces (see Supporting Information Note 1 and Figure S1) that are smooth and chemically homogeneous (see Figure S2). Figure 1a shows a bright-field (BF) transmission electron microscopy (TEM) image of the Mo_2_C film cross section. The film is very homogeneous in thickness and shows different areas of ordered growth with changes in the crystallite orientation depending on the underlying substrate grains. The high-resolution (HR) TEM image (inset in Figure 1a), together with the selected area electron diffraction (SAED) pattern, reveal the best agreement of our material’s phase with hexagonal Mo_2_C (see Figures S3 and S4). Details of the evaluation of the structural data are given in Supporting Information Note 1.

To get a macroscopic picture of the electrocatalytic activity of this planar hexagonal Mo_2_C film electrode, cyclic voltammograms (CVs) were recorded in 0.1 M KHCO_3_ with CO_2_ and 0.1 M NaClO_4_ without CO_2_ (both pH 6.9) over the potential (*E*) region of interest (see Figure 1b and Supporting Information Note 2). These electrolytes were selected to have a minimized interaction of ions with the interface and for the meaningful comparison of the Mo_2_C electrochemistry with and without CO_2_ at the same pH. After immersion under potential control at −0.70 *V*_SHE_, the potential was cycled between −1.6 and −0.2 *V*_SHE_. A current density of ca. −20 mA cm^–2^ is reached at −1.6 *V*_SHE_ when CO_2_ is present. Notably, care has to be taken to not apply potentials more positive than −0.2 *V*_SHE_ due to the danger of anodically oxidizing the electrodes, as indicated by the positive current peak (independently recorded CV, dashed blue line) visible in Figure 1b. The cathodic current is due to the reduction of neutral water and shows the same shape for both electrolytes. With CO_2_ present, the onset of the reduction is slightly shifted to less negative potentials, which we attribute to the presence of carbonate and bicarbonate species (see below).

To qualitatively identify the reaction products forming upon cathodic polarization, differential electrochemical mass spectrometry (DEMS, Figure S5) has been employed. No valuable CO_2_ reduction product, such as methane (CH_4_) or ethylene (C_2_H_4_), but exclusively hydrogen was detected under the conditions applied in this work at pH 6.9. This means that the whole potential range of interest is governed by the HER. To guarantee the detectability of all products of interest, a reference measurement was conducted with a polycrystalline Cu sample, where CH_4_ and C_2_H_4_ were indeed found at potentials <−1.2 *V*_SHE_. At a different pH of 3.7, gas chromatography also confirmed the exclusive formation of hydrogen over our Mo_2_C films in the complete cathodic potential range (see Supporting Information Note 2). The HER is enhanced when buffering CO_2_/carbonate species are present in the electrolyte, and it is this enhancement, not any CO_2_ reduction, that stands behind the slight shift of the onset potential seen in Figure 1b.

### 2.2 *Ab Initio* Thermodynamics of Mo_2_C in Aqueous Electrolyte

Unexpectedly, no CO_2_ electroreduction is thus found upon cathodic polarization of this heralded compound material. One immediate explanation for this could be the material’s known susceptibility to oxidation. However, in full agreement with the experimental Pourbaix diagram of Mo_2_C,^25^ bulk oxidation is only clearly remarkable at ∼0.04 *V*_SHE_ at pH 6.9 (Figure 1b), a potential which we deliberately never applied in the above studies. In principle, one would also expect residing surface oxides or oxygenated adsorbates to be cathodically reduced during the highly negative potentials applied for the CO_2_ reduction. On the other hand, a sometimes surprisingly large extra thermodynamic stability of surface oxides has been reported in thermal oxidation catalysis.^31,32^

**Figure 2:**
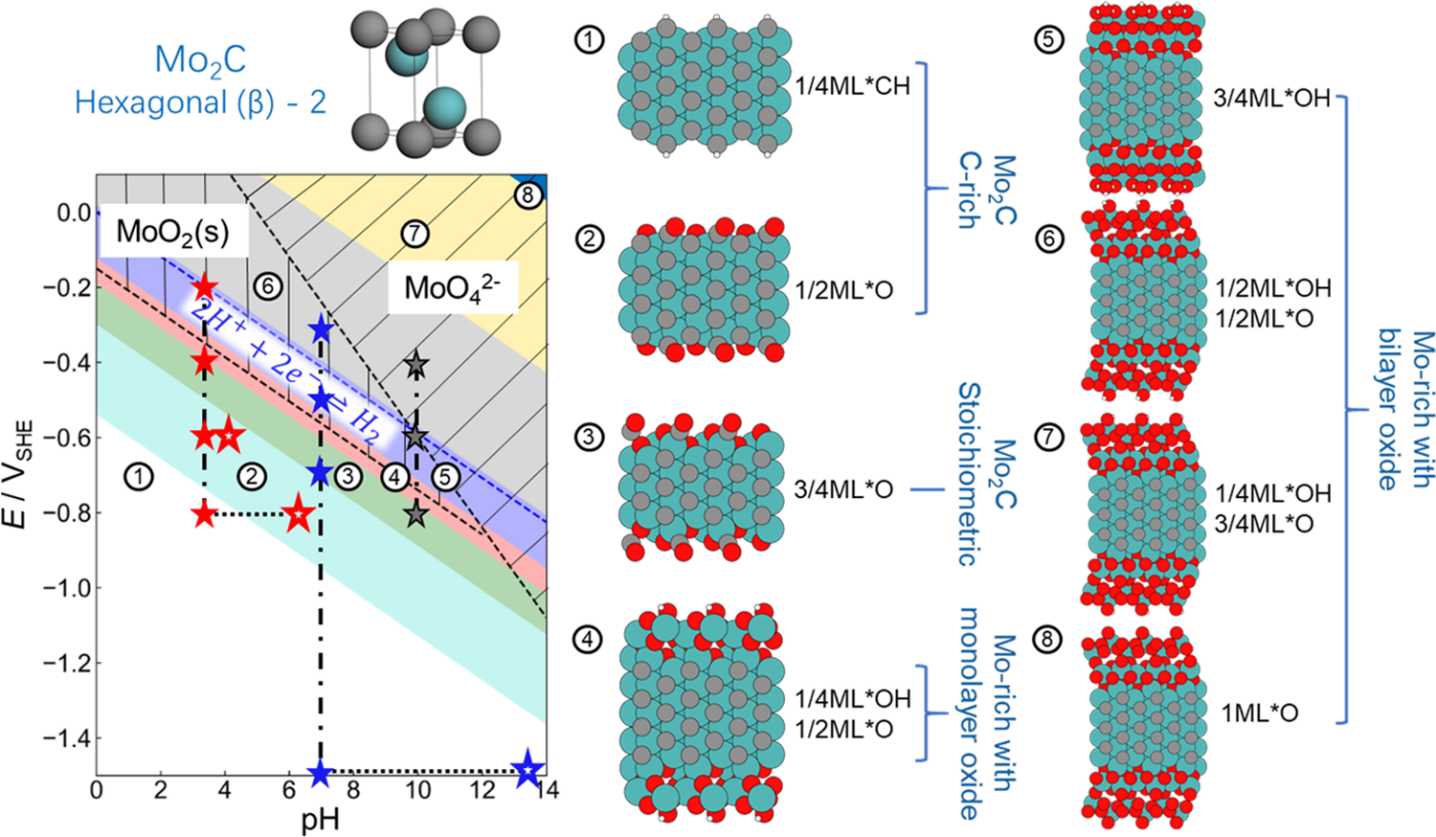
*Ab initio* thermodynamics surface Pourbaix (*E*/pH) diagram for Mo_2_C(110) and possible MoO_2_(100) surface oxide formation. The data here are for the more stable hexagonal (β)-2 bulk phase, while analogue results for the (β)-1 phase are given in Figure S6. The generic stability ranges of bulk MoO_2_ and dissolved MoO_4_^2–^, as calculated for the parent metal,^27^ are indicated by black hatched areas. Side views of the stable surface phases ①–⑧ are shown on the right (Mo, C, O, and H atoms are depicted as green, gray, red, and white spheres, respectively). The surface terminations in terms of fractions of monolayers (ML) defined with respect to MoO_2_(100)-(1 × 1) are also provided. The blue dashed line indicates the thermodynamic onset potential of the hydrogen evolution reaction. Experimentally tested conditions are marked by stars, solid stars for nominal pH conditions, and hollow stars after considering surface pH changes (see text and Figure 4).

To assess this for the present case, we performed DFT-based ab initio thermodynamics to determine the stable surface phases of the dominant Mo_2_C(110) facet in aqueous electrolyte (see Supporting Information Note 3). We computed the surface Pourbaix diagrams for two different bulk phases, each time considering more than 300 candidate structures of pristine Mo_2_C terminations with or without coincidence and minimally strained MoO_2_(100) surface oxide films of varying thickness, and with varying degrees of hydrogenation or hydroxylation. The resulting surface Pourbaix diagram for the (more stable) hexagonal close-packed (β)-2 bulk phase is depicted in Figure 2. The surface Pourbaix diagram of the (β)-1 phase is given in Figure S6 and leads to analogue conclusions. Eight different stable surface phases emerge in the relevant *E*/pH range. At extremely negative potentials and low pH values, a carbon-rich Mo_2_C termination with a low amount of adsorbed hydrogen prevails (see region ① in Figure 2). Oxygen or hydroxyl surface films dominate in the less reducing regions ② and ③, followed by a slightly hydroxylated monolayer (ML) thick surface oxide in region ④. An oxide bilayer with a varying degree of hydroxylation finally forms in regions ⑤–⑧.

Not surprisingly, the surface thus changes with decreasing *E* and pH from an oxidized to a reduced state with a still partially hydrogenated carbon-rich Mo_2_C termination. Intriguingly however, this transition of the surface chemistry happens at much more negative potentials than expected from the experimental bulk Pourbaix diagram of Mo_2_C,^25^ i.e., higher overpotentials are required to reduce the surface. Comparing the experimental apparent bulk stability and theoretical surface stability lines (Figure S7) suggests that the overpotential to theoretically reach a Mo_2_C surface free of oxygen-containing adsorbates is in fact almost pH-independent and as high as ∼1.0 V. This implies that the benefit of a possibly suitable reactive carbide surface chemistry for CO_2_ electroreduction will only become accessible under conditions where the competing HER already proceeds at extremely high overpotentials (see blue dashed line in Figure 2 for the HER onset). Different from the bare Mo_2_C surface,^7^ MoO_2_ is a recognized HER catalyst.^33−35^ Free-energy calculations for the first protonation step of the HER and CO_2_RR summarized in Figure S8 indeed indicate a high selectivity toward the HER also for the Mo_2_C surface covered with a MoO_2_ bilayer, i.e., structure ⑧ in Figure 2. This rationalizes the dominant hydrogen evolution seen in our electrocatalytic measurements and casts severe doubts on a promising activity toward the CO_2_RR that could be reached with Mo_2_C catalysts in aqueous electrolyte.

### 2.3 Experimental Surface Pourbaix Diagram

While the ab initio thermodynamics results thus provide a sound rationalization for the initially surprising inactivity toward the CO_2_RR, one has to stress that the absolute position of stability lines in computed Pourbaix diagrams depends sensitively on the employed DFT functional.^32^ The surface Pourbaix diagram was furthermore only computed for the dominant Mo_2_C(110) facet, and the formation of a coincidence surface oxide film was assumed. Before reaching a final conclusion regarding a future use of Mo_2_C as a CO_2_RR catalyst, we therefore scrutinize the obtained picture with quasi *in-situ* X-ray photoelectron spectroscopy (XPS).

### 2.4 Mo_2_C Film Analysis after Its Synthesis

**Figure 3:**
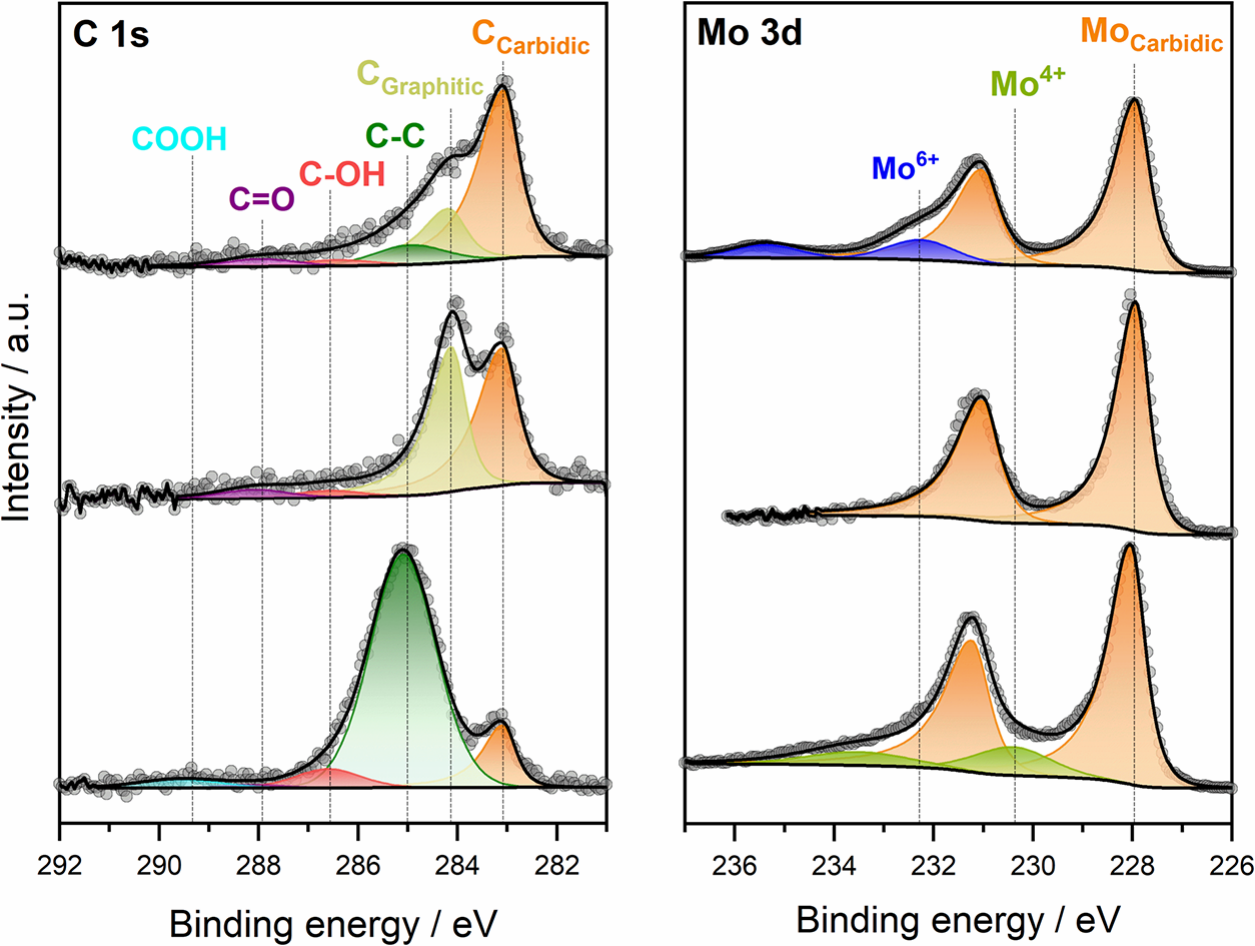
Surface chemistry of Mo_2_C showing MoO_3_ after air exposure and MoO_2_ after electrolyte contact at reductive potentials. Deconvoluted X-ray photoelectron C 1s (left) and Mo 3d (right) high-resolution spectra of the freshly synthesized film (top) with native MoO_3_ after short (15 min) air contact, (middle) without oxide due to handling under hydrogen and in an Ar-filled glovebox at all times, and (bottom) with MoO_2_ after immersion of oxide-free Mo_2_C into 0.1 M NaClO_4_ (pH 3.7) at −0.2 *V*_SHE_ for 15 min. The fit components are directly given in the figure; the takeoff angle is 0° in all cases.

We started our analysis of the exact chemical composition in terms of existing oxidation states and stoichiometry before and after cathodic polarization with the pristine Mo_2_C films, directly after their synthesis. Here, we first handled the Mo_2_C without protection from ambient air. Importantly, due to air exposure, adventitious carbon and oxidized Mo species are always detected at the Mo_2_C surface; an example of the typical surface chemistry is shown in Figure 3 (top panels). The same behavior has been observed in the case of titanium oxycarbide surfaces and is very likely related to the low intrinsic thermodynamic stability of the material at ambient oxygen pressures.^36^ The amount of oxide can vary slightly and depends on the exact synthesis conditions (details are given in Table S2). No other undesirable element is found, which confirms the chemical purity of the synthesized Mo_2_C specimen (see Figure S9).

The fit of the XPS spectra of Mo_2_C after a 15 min air contact in Figure 3 (top) reveals contributions from MoO_3_, graphitic, and adventitious carbon species, while the primary components are carbidic Mo and carbidic carbon. Details of the evaluation and fitting of the spectra are given in Supporting Information Note 4. The average thickness determined for the native oxide surface film lies between 0.6 and 0.7 nm (see Figures S10 and S14), which fits nicely to the expected thickness of one double layer of edge-shared MoO_6_ octahedra constituting layered MoO_3_.^37^ Carbon and oxide can be easily removed from the surface through 60 s of Ar sputtering in the XPS (Figure S12). It has to be noted that the synthesis of oxide-free stoichiometric Mo_2_C is possible in a reactor where the Mo_2_C specimen can be transported protected under Ar and/or H_2_ gas (see Figure 3 (middle) and Table S4). Systematic contact to air leads to surface oxide (MoO_3_) formation within less than 15 min (see Figure S15).

Importantly, exposure of the oxide-free carbide to aqueous electrolyte also leads to the spontaneous formation of an oxide overlayer, even if reducing conditions are applied through immersion at −0.2 *V*_SHE_ into the most acidic electrolyte (see Figure 3, bottom). After contact of the specimen with electrolyte, the sole detectable oxides are MoO_2_ and/or nonstoichiometric suboxides (see Figure 3 and Table S5 and explanation in the next paragraph). This insight is central to the application of oxidation-prone carbide materials in electrocatalysis in general, because it reveals the sheer impossibility to start from an oxide- or oxygen-free catalyst/electrolyte interface, which has not been clearly recognized in the past. The only conceivable way to remove the oxide from the surface could therefore be the polarization at extremely negative potentials in regions where massive HER activity is expected. However, in view of the concomitant surface pH effects described below, it might be that electrochemical surface oxide reduction is not practically feasible at all.

### 2.5 Mo_2_C Surface Chemistry after Cathodic Polarization

**Figure 4:**
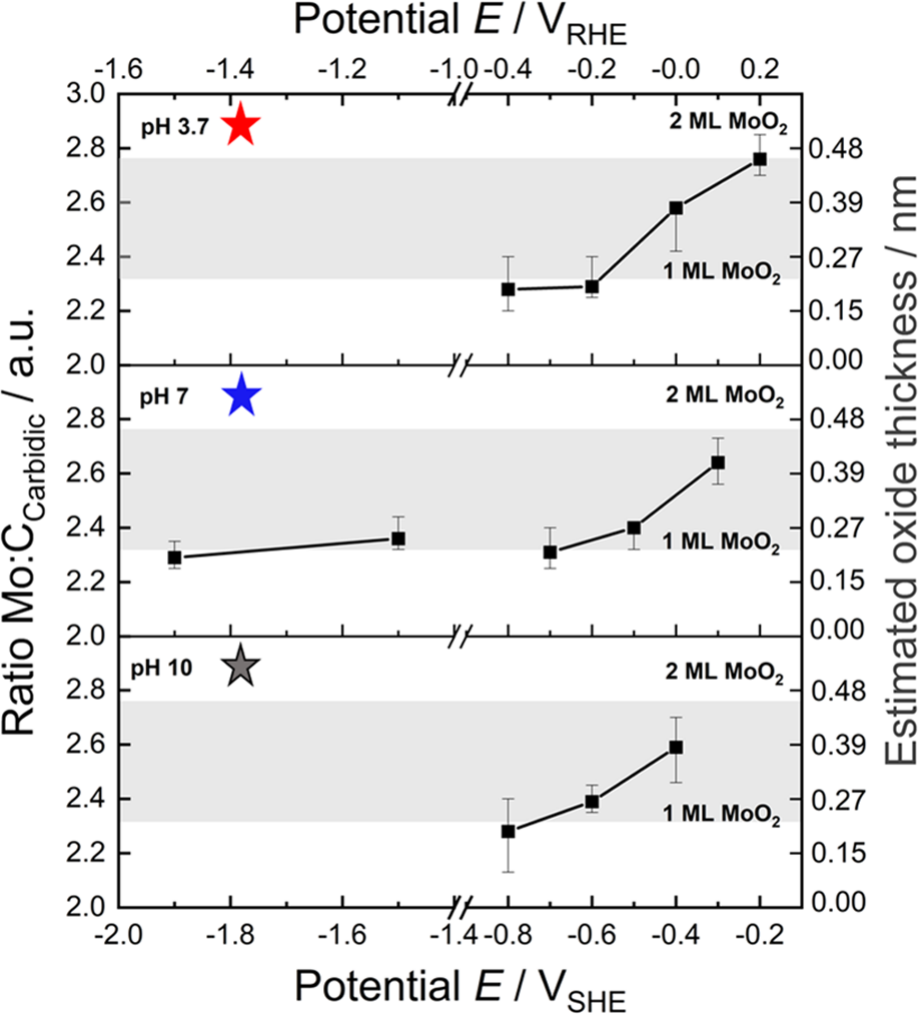
Experimental Pourbaix diagram confirming surface oxide formation under all relevant CO_2_RR conditions. The correlation between the Mo/C_carbidic_ ratio and the estimated oxide layer thickness is given in Figure S19. For the calculation of the error bars, we refer to Supporting Information Note 4. The shaded regions indicate the thickness expected for one or two monolayers (ML) MoO_2_(100). The probed reaction conditions are indicated by correspondingly colored stars in the theoretical surface Pourbaix diagram in Figure 2.

To investigate the surface chemistry of the Mo_2_C films after electrolyte exposure at specific applied potentials, quasi *in-situ* XPS studies were carried out with the help of an air-tight transfer vessel for specimen transport from the Ar-filled glovebox, where the electrochemistry was carried out, to the XPS, where a second transfer under Ar was undertaken as described in ref. (38) (see Figure S16). Different *E*/pH values were selected to monitor changes of the electrode surface after cathodic polarization in 0.1 M NaClO_4_ solution. The potential steps were individually selected for three specific pH values of 3.7, 7, and 10, based on central points in the theoretical surface Pourbaix diagram. The corresponding CVs are shown in Figure S17. After potentiostatic polarization, XPS spectra (Figure S18) were recorded to calculate the Mo/C_carbidic_ ratio and estimate the oxide layer thicknesses shown in Figure 4. The oxide overlayer exclusively consists of MoO_2_ and/or, as the case may be, suboxides. This suggests that MoO_3_ gets dissolved upon electrode immersion, which agrees well with the bulk Pourbaix diagram of the Mo/MoO*_x_* system.^26,27,39^ Therefore, for the estimation of the surface oxide layer thickness, it was assumed that the film only consists of MoO_2_. A detailed description of the data evaluation assuming uniform oxide distribution (complete “wetting”) is given in Supporting Information Note 4 and Figure S19.

At pH 3.7, polarization at −0.2 *V*_SHE_ leads to the formation of an ∼0.46 nm thick film, which corresponds almost perfectly to the thickness of 0.48 nm of a bilayer MoO_2_(100) predicted to form atop the Mo_2_C under these conditions in the theoretical surface Pourbaix diagram (region ⑤ in Figure 2). Stepping to more negative potentials at pH 3.7 results in a decrease of the oxide film thickness down to ∼0.20 nm at ≤−0.6 *V*_SHE_, which would then correspond to roughly one or below one monolayer coverage, again exactly as expected from the theoretical surface Pourbaix diagram. A similar evolution from about bilayer surface oxide to below monolayer surface oxide is found upon increasing cathodic polarization at the other two probed pH values 7 and 10. In the case of pH 7, this oxide is stable down to potentials as low as −1.9 *V*_SHE_. As further discussed below, all of these findings are fully consistent with the theoretical surface Pourbaix diagram; see the stars in Figure 2 marking the location of the probed reaction conditions.

For comparison with realistic CO_2_RR conditions, additional studies have been carried out in 0.1 M CO_2_-saturated KHCO_3_ (pH 6.9). After polarization at −0.4 and −1.5 *V*_SHE_, thin surface oxide films with thicknesses in the same range as those determined for 0.1 M NaClO_4_ (pH 7) are detected with XPS (see Figure S20 and Tables S9–S11).

### 2.6 Surface pH Correction

The overall consistency of the experimental and theoretical Pourbaix diagram is truly remarkable considering not least the known uncertainty in computed absolute stability regions.^32^ Nevertheless, it is intriguing to note that at pH 3.7, our experiments still indicate the presence of a surface oxide of about 1 ML thickness even at the most negative potential probed. Under such reducing conditions, the intrinsic state of the surface should have just been reached according to theory. To further analyze this, we extended the measurements to very negative potentials, specifically for pH 7, as this is the sole pH where meaningful CO_2_ electroreduction is possible in aqueous electrolytes. Surprisingly, these measurements also indicate the stability of about 1 ML thick surface oxide even at a polarization as negative as −1.9 *V*_SHE_ (see Figure 4), where theory would predict the bare carbide surface beyond doubt (see Figure 2).

This discrepancy can be resolved by recognizing that non-negligible water and/or proton reduction currents flow at these negative potentials (Figure S21 and Table S12). This can give rise to significant changes in the surface pH due to mass transfer limitations, i.e., the actual pH at the carbide/liquid interface could differ strongly from the nominal bulk pH set in the experiments. To assess this, we calculated the approximate surface pH that occurs during the potentiostatic experiments solving a simplified Poisson–Nernst–Planck (PNP) transport model^40^ (see Supporting Information Note 4, Figures S22–S24, and Table S13 for a detailed account). Indeed, these calculations confirm strong changes, which, for instance, at pH 7 and −1.5 *V*_SHE_ lead to an effective pH shift to strongly alkaline conditions (pH 13–14) in front of the electrode. The real reaction conditions thus probed in the experiments are indicated by hollow stars in Figure 2 and are now fully consistent with the theoretical predictions within the expected uncertainties. Note that we did not explicitly engage in a calculation of the pH shift for the most negative −1.9 *V*_SHE_ at pH 7, as bubble formation and a likely ion deposition from the electrolyte under the then highly violent HER is not captured by the simple PNP transport model anymore.

### 2.7 CO_2_ Electroreduction in Nonaqueous Electrolyte

The analyses presented so far unfortunately confirm that the TMC’s theoretically predicted promising CO_2_RR chemistry is not experimentally accessible in aqueous electrolytes due to the persistent formation of a thin oxidic film at the surface. Truly harvesting this chemistry thus requires to move to alternative approaches that prevent such passivation. As shown in Figure 3, it is possible to avoid oxidation after synthesis, to transfer the electrode to the electrochemical cell without any contact to ambient air, and to immerse it under potential control. The last consequent measure to prevent operando oxidation is then to switch to a nonaqueous electrolyte.

**Figure 5:**
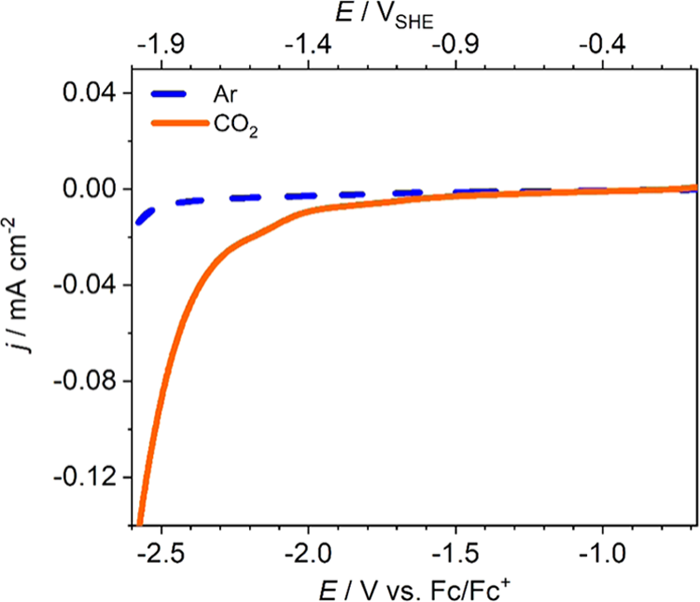
CO_2_ electroreduction at Mo_2_C in nonaqueous electrolyte. CVs of Mo_2_C without (blue) and with (orange) CO_2_ in acetonitrile with 0.1 M tetrabutylammonium hexafluorophosphate (TBAPF_6_). Scan rate: 20 mV s^–1^.

Recognizing that CO_2_RR in nonaqueous electrolytes is a widely unchartered territory, we carried out corresponding proof-of-principle experiments in acetonitrile-based electrolyte that only contains traces of water (see Supporting Information Note 5 and Table S14). As shown in Figure 5, this immediately leads to a pronounced reduction current in the presence of CO_2_ with an approximate onset at −1.7 V versus the ferrocene/ferrocenium (Fc/Fc^+^) redox couple (calibration in Figure S25) and thus roughly at −1.08 *V*_SHE_.^41^ Polycrystalline Cu shows an approximate CO_2_ reduction onset at −1.31 *V*_SHE_ in the same electrolyte.^42^ A current density of 0.1 mA cm^–2^ is reached at a potential of ≤−2.52 *V*_Fc/Fc^+^_ (≤−1.9 *V*_SHE_). In contrast, no reduction peak is visible in Ar-purged electrolyte, where no CO_2_ is present. First electrochemical infrared spectroscopy data indicate that CO forms as a product at potentials ≤−2.5 *V*_Fc/Fc^+^_ (≤−1.9 *V*_SHE_) (see Figure S26). This preliminary insight confirms that under conditions that preserve the Mo_2_C chemistry, the material indeed shows high CO_2_ electroreduction activity as had been predicted in the earlier computational screening studies.^20,21^ This immediate success now motivates follow-up work that analyzes the formed reaction products and systematically explores the activity toward the CO_2_RR of this and other TMCs in nonaqueous electrolytes.

## 3 Conclusions

In summary, we have demonstrated that well-defined planar hexagonal Mo_2_C films exhibit the intrinsic property to passivate at their surface upon air exposure and immersion in aqueous electrolyte, even under reductive conditions and regardless if they are native oxide-free or already passivated before contact with the electrolyte. After immersion in aqueous electrolyte with pHs 3.7, 7, and 10, monolayer-thin MoO_2_-like oxidic films are present at the surface that are stable down to potentials as negative as <−1.9 *V*_SHE_ at pH 7. The oxide-covered carbides exclusively show activity toward water reduction, and no CO_2_ electroreduction products are found. The presence of CO_2_ in the electrolyte only enhances the HER, likely through changes of the surface pH and/or transport of protons by buffer ions.

This means that the promising CO_2_RR properties of this TMC heralded by earlier computational studies^7,20,21^ are experimentally not accessible in aqueous electrolytes. Quite discomfortingly, the bulk Pourbaix diagrams of other prominent carbides like W_2_C or WC,^25^ as well as the bulk Pourbaix diagrams of important parent metals like V, Zr, or Nb,^27^ suggest a similar propensity to oxidation as molybdenum. A possible operando formation of ultrathin surface oxidic films was generally not considered in previous screening work on TMCs. Our results thus dictate to revisit the suitability of this materials class for CO_2_RR from this perspective. Alternatively, first experiments with acetonitrile indicate that a switch to nonaqueous base electrolytes (with only controlled water addition as a proton source) circumvents this issue and indeed confirms Mo_2_C as an efficient CO_2_RR catalyst.

## 4 Experimental Section

### 4.1 Synthesis of Mo_2_C Films

A detailed description of the applied synthesis route is given in Supporting Information Note 6.

### 4.2 Surface Characterization

#### 4.2.1 Microscopy

The surface morphology of the synthesized Mo_2_C was investigated with atomic force microscopy (AFM) and scanning electron microscopy coupled with energy-dispersive X-ray spectroscopy (SEM–EDX). Transmission electron microscopy (TEM) was used for cross-sectional characterization. Details are outlined in Supporting Information Note 1.

#### 4.2.2 X-ray Photoelectron Spectroscopy (XPS)

XPS spectra were acquired on a MultiLab 2000 instrument (Thermo Fisher Scientific) comprising a hemispherical sector analyzer (Alpha 110, Thermo Fisher Scientific) and a monochromated X-ray source (Al K_α_, 1486.6 eV). A flood gun was used for charge compensation via emission of electrons at a kinetic energy of 6 eV, and charge-induced shifts were corrected with reference to the carbidic carbon component at 283.1 eV. Ar sputtering (3 keV, ion current of 1.0 μA) was used for depth profiling by scanning across an area of (1.5 × 1.5) mm^2^. Nondestructive depth profiling was carried out by adjusting the angle between the sample and the analyzer (0–60°). Survey scans were obtained at a pass energy of 100 eV with an energy step size of 1 eV. XPS spectra of the binding energy (BE) regions of interest (C 1s, O 1s, Mo 3d) were recorded at a pass energy of 25 eV and an energy step size of 0.1 eV. Peak fitting was processed with CasaXPS software,^43^ always using a Shirley-type background and mixed Gaussian–Lorentzian (GL30) functions except for the carbide peaks, where asymmetric peak shapes were utilized. The quantification of all spectra was corrected for different escape depths via the Gries^44^ formula.

### 4.3 Electrochemistry

All electrochemistry measurements were carried out at room temperature in aqueous 0.1 M Ar-saturated NaClO_4_ and CO_2_-saturated KHCO_3_ or in nonaqueous acetonitrile solutions containing 0.1 M tetrabutylammonium hexafluorophosphate (TBAPF_6_) using an Autolab (Metrohm) or a BioLogic (VMP3, BioLogic) potentiostat. Details are outlined in Supporting Information Notes 2 and 5.

#### 4.3.1 Quasi *In-Situ* XPS Studies

The Mo_2_C electrodes were potentiostatically polarized in a glovebox (MB 200B Eco, MBraun) using a commercial three-electrode glass cell with a flame-annealed carbon rod (Ultra Carbon Corporation) counter electrode and an activated carbon quasi-reference electrode.^45^ Selected potentials were applied for 15 min, followed by emersion under potential control and flushing with deionized water. Afterward, the specimen was transported to the XPS analyzing chamber in a home-built transfer cell without exposure to ambient for characterization of their surface chemistries. The transfer cell is depicted in Figure S16.

### 4.4 Computational Details

#### 4.4.1 *Ab Initio* Thermodynamics

The Pourbaix diagram is calculated by evaluating the surface free energy γ for a range of candidate structures and identifying the most stable surface composition or geometry as the one that minimizes γ at any given bias *E* and pH. In-depth information on these calculations is provided in Supporting Information Note 3.

#### 4.4.2 DFT Calculations

All DFT calculations (see also Supporting Information Note 3) were performed with the plane-wave DFT code QuantumESPRESSO (QE)^46^ using the van der Waals-corrected BEEF-vdW exchange correlation functional.^47^ The basis for all surface candidate structures consists of a seven-layer Mo_2_C slab, to which oxide layers were added at both sides. A vacuum layer of at least 15 Å ensured a decoupling of consecutive slabs in the supercell geometry. A (1 × 2) MoO_2_(110) surface unit cell allowed to test O, H, and/or OH concentration variations in 1/4 ML steps. The corresponding surface unit-cell areas *A* = 27.30 Å^2^ for bulk phase (β)-1 (Figure S6) and 24.88 Å^2^ for phase (β)-2 (Figure 2) result from the optimized bulk lattice constants of these two phases of hexagonal Mo_2_C. Using a plane-wave cutoff of 800 eV and a (4 × 4 × 1) k-grid, all structures were fully relaxed until residual forces fell below 0.03 eV/Å, while keeping the middle three Mo_2_C slab layers in their frozen bulklike positions. Test calculations with higher cutoffs and k-point grids indicate the obtained surface free energies to be converged within 10 and 1 meV/Å^2^, respectively. Solvation effects were considered within an implicit solvation approach using the Environ module provided with QE.^48^ The relative permittivity value of the dielectric layer was set to 78.3 to simulate water. The DFT calculations for the bulk and molecular reservoirs were performed as follows: The bulk hexagonal phase Mo_2_C was calculated with a (1 × 1 × 1) unit cell and a (12 × 12 × 12) k-grid. The gas-phase molecules (H_2_, H_2_O, and CO_2_) were calculated for electronic energies and vibrational frequencies separately in each supercell with a side length of 10 Å.

## Supporting Information Available

Additional characterization (AFM, SEM, TEM, XPS); product analysis (DEMS, GC, IR); additional ab initio thermodynamic calculations; fitted XPS spectra; XPS fitting parameters; determination of Mo_2_C and its native oxide film thicknesses; current transients; pH correction calculations; and Karl–Fischer titration results (PDF)

## References

[ref1] Nørskov J. K.; Bligaard T.; Rossmeisl J.; Christensen C. H. Towards the Computational Design of Solid Catalysts. Nat. Chem. 2009, 1, 37–46. 10.1038/nchem.121. 21378799

[ref2] Greeley J. Theoretical Heterogeneous Catalysis: Scaling Relationships and Computational Catalyst Design. Annu. Rev. Chem. Biomol. Eng. 2016, 7, 605–635. 10.1146/annurev-chembioeng-080615-034413. 27088666

[ref3] Zhao Z.-J.; Liu S.; Zha S.; Cheng D.; Studt F.; Henkelman G.; Gong J. Theory-Guided Design of Catalytic Materials Using Scaling Relationships and Reactivity Descriptors. Nat. Rev. Mater. 2019, 4, 792–804. 10.1038/s41578-019-0152-x.

[ref4] Védrine J. Heterogeneous Catalysis on Metal Oxides. Catalysts 2017, 7, 341 10.3390/catal7110341.

[ref5] Li Z.; Wu Y. 2D Early Transition Metal Carbides (MXenes) for Catalysis. Small 2019, 15, 1804736 10.1002/smll.201804736. 30672102

[ref6] Wan W.; Tackett B. M.; Chen J. G. Reactions of Water and C1 Molecules on Carbide and Metal-Modified Carbide Surfaces. Chem. Soc. Rev. 2017, 46, 1807–1823. 10.1039/C6CS00862C. 28229154

[ref7] Li H.; Reuter K. Active-Site Computational Screening: Role of Structural and Compositional Diversity for the Electrochemical CO_2_ Reduction at Mo Carbide Catalysts. ACS Catal. 2020, 10, 11814–11821.

[ref8] Levy R. B.; Boudart M. Platinum-Like Behavior of Tungsten Carbide in Surface Catalysis. Science 1973, 181, 547–549. 10.1126/science.181.4099.547. 17777803

[ref9] Weigert E. C.; Stottlemyer A. L.; Zellner M. B.; Chen J. G. Tungsten Monocarbide as Potential Replacement of Platinum for Methanol Electrooxidation. J. Phys. Chem. C 2007, 111, 14617–14620. 10.1021/jp075504z.

[ref10] Rodriguez J. A.; Illas F. Activation of Noble Metals on Metal-Carbide Surfaces: Novel Catalysts for CO Oxidation, Desulfurization and Hydrogenation Reactions. Phys. Chem. Chem. Phys. 2012, 14, 427–438. 10.1039/C1CP22738F. 22108864

[ref11] Hunt S. T.; Nimmanwudipong T.; Román-Leshkov Y. Engineering Non-Sintered, Metal-Terminated Tungsten Carbide Nanoparticles for Catalysis. Angew. Chem., Int. Ed. 2014, 53, 5131–5136. 10.1002/anie.201400294. 24700729

[ref12] Huo C.-F.; Li Y.-W.; Wang J.; Jiao H. Insight into CH_4_ Formation in Iron-Catalyzed Fischer–Tropsch Synthesis. J. Am. Chem. Soc. 2009, 131, 14713–14721. 10.1021/ja9021864. 19780531

[ref13] Liu P.; Rodriguez J. A. Water-Gas-Shift Reaction on Molybdenum Carbide Surfaces: Essential Role of the Oxycarbide. J. Phys. Chem. B 2006, 110, 19418–19425. 10.1021/jp0621629. 17004800

[ref14] Dubois J.-L.; Sayama K.; Arakawa H. CO_2_ Hydrogenation over Carbide Catalysts. Chem. Lett. 1992, 1, 5–8. 10.1246/cl.1992.5.

[ref15] Medford A. J.; Vojvodic A.; Studt F.; Abild-Pedersen F.; Nørskov J. K. Elementary Steps of Syngas Reactions on Mo_2_C(001): Adsorption Thermochemistry and Bond Dissociation. J. Catal. 2012, 290, 108–117. 10.1016/j.jcat.2012.03.007.

[ref16] Gracia J. M.; Prinsloo F. F.; Niemantsverdriet J. W. Mars-van Krevelen-like Mechanism of CO Hydrogenation on an Iron Carbide Surface. Catal. Lett. 2009, 133, 257–261. 10.1007/s10562-009-0179-5.

[ref17] Dhandapani B.; St. Clair T.; Oyama S. T. Simultaneous Hydrodesulfurization, Hydrodeoxygenation, and Hydrogenation with Molybdenum Carbide. Appl. Catal., A 1998, 168, 219–228. 10.1016/S0926-860X(97)00342-6.

[ref18] Ren H.; Hansgen D. A.; Stottlemyer A. L.; Kelly T. G.; Chen J. G. Replacing Platinum with Tungsten Carbide (WC) for Reforming Reactions: Similarities in Ethanol Decomposition on Ni/Pt and Ni/WC Surfaces. ACS Catal. 2011, 1, 390–398. 10.1021/cs200057y.

[ref19] Vojvodic A. Steam Reforming on Transition-Metal Carbides from Density-Functional Theory. Catal. Lett. 2012, 142, 728–735. 10.1007/s10562-012-0820-6.

[ref20] Michalsky R.; Zhang Y.-J.; Medford A. J.; Peterson A. A. Departures from the Adsorption Energy Scaling Relations for Metal Carbide Catalysts. J. Phys. Chem. C 2014, 118, 13026–13034. 10.1021/jp503756g.

[ref21] Peterson A. A.; Nørskov J. K. Activity Descriptors for CO_2_ Electroreduction to Methane on Transition Metal Catalysts. J. Phys. Chem. Lett. 2012, 3, 251–258. 10.1021/jz201461p.

[ref22] Li N.; Chen X.; Ong W.-J.; MacFarlane D. R.; Zhao X.; Cheetham A. K.; Sun C. Understanding of Electrochemical Mechanisms for CO_2_ Capture and Conversion into Hydrocarbon Fuels in Transition-Metal Carbides (MXenes). ACS Nano 2017, 11, 10825–10833. 10.1021/acsnano.7b03738. 28892617

[ref23] Kortlever R.; Shen J.; Schouten K. J. P.; Calle-Vallejo F.; Koper M. T. M. Catalysts and Reaction Pathways for the Electrochemical Reduction of Carbon Dioxide. J. Phys. Chem. Lett. 2015, 6, 4073–4082. 10.1021/acs.jpclett.5b01559. 26722779

[ref24] Kim S. K.; Zhang Y.-J.; Bergstrom H.; Michalsky R.; Peterson A. Understanding the Low-Overpotential Production of CH_4_ from CO_2_ on Mo_2_C Catalysts. ACS Catal. 2016, 2003–2013. 10.1021/acscatal.5b02424.

[ref25] Weidman M. C.; Esposito D. V.; Hsu Y.-C.; Chen J. G. Comparison of Electrochemical Stability of Transition Metal Carbides (WC, W_2_C, Mo_2_C) over a Wide pH Range. J. Power Sources 2012, 202, 11–17. 10.1016/j.jpowsour.2011.10.093.

[ref26] Deltombe E.; Pourbaix M. Atlas of Electrochemical Equilibria in Aqueous Solutions; National Association of Corrosion Engineers, 1974; p 272.

[ref27] Takeno N. Atlas of E-pH Diagrams: Intercomparison of Thermodynamic Databases, Geological Survey of Japan Open File Report No. 419; Research Center for Deep Geological Environments, 2005.

[ref28] Foelske-Schmitz A. X-ray Photoelectron Spectroscopy in Electrochemistry Research. In Encyclopedia of Interfacial Chemistry; Elsevier, 2018; pp 591–606.

[ref29] Auer A.; Andersen M.; Wernig E.; Hörmann N. G.; Buller N.; Reuter K.; Kunze-Liebhäuser J. Self-Activation of Copper Electrodes during CO Electro-Oxidation in Alkaline Electrolyte. Nat. Catal. 2020, 3, 797 10.1038/s41929-020-00505-w.

[ref30] Bruix A.; Margraf J. T.; Andersen M.; Reuter K. First-Principles-Based Multiscale Modelling of Heterogeneous Catalysis. Nat. Catal. 2019, 2, 659–670. 10.1038/s41929-019-0298-3.

[ref31] Campbell C. T. Transition Metal Oxides: Extra Thermodynamic Stability as Thin Films. Phys. Rev. Lett. 2006, 96, 066106 10.1103/PhysRevLett.96.066106. 16606017

[ref32] Reuter K. Ab Initio Thermodynamics and First-Principles Microkinetics for Surface Catalysis. Catal. Lett. 2016, 146, 541–563. 10.1007/s10562-015-1684-3.

[ref33] Chen X.; Liu G.; Zheng W.; Feng W.; Cao W.; Hu W.; Hu P. Vertical 2D MoO_2_/MoSe_2_ Core-Shell Nanosheet Arrays as High-Performance Electrocatalysts for Hydrogen Evolution Reaction. Adv. Funct. Mater. 2016, 26, 8537–8544. 10.1002/adfm.201603674.

[ref34] Jian C.; Cai Q.; Hong W.; Li J.; Liu W. Edge-Riched MoSe_2_/MoO_2_ Hybrid Electrocatalyst for Efficient Hydrogen Evolution Reaction. Small 2018, 14, 1703798 10.1002/smll.201703798. 29399992

[ref35] Zeng H.; Chen S.; Jin Y. Q.; Li J.; Song J.; Le Z.; Liang G.; Zhang H.; Xie F.; Chen J.; Jin Y.; Chen X.; Meng H. Excellent Hydrogen Evolution and Oxidation Activities in Acid. ACS Energy Lett. 2020, 5, 1908–1915. 10.1021/acsenergylett.0c00642.

[ref36] Calvillo L.; Fittipaldi D.; Rüdiger C.; Agnoli S.; Favaro M.; Valero-Vidal C.; Di Valentin C.; Vittadini A.; Bozzolo N.; Jacomet S.; Gregoratti L.; Kunze-Liebhäuser J.; Pacchioni G.; Granozzi G. Carbothermal Transformation of TiO_2_ into TiO_x_C_y_ in UHV: Tracking Intrinsic Chemical Stabilities. J. Phys. Chem. C 2014, 118, 22601–22610. 10.1021/jp506728w.

[ref37] Floquet N.; Bertrand O.; Heizmann J. J. Structural and Morphological Studies of the Growth of MoO_3_ Scales during High-Temperature Oxidation of Molybdenum. Oxid. Met. 1992, 37, 253–280. 10.1007/BF00665191.

[ref38] Watschinger M.; Ploner K.; Winkler D.; Kunze-Liebhäuser J.; Klötzer B.; Penner S. Operando Fourier-Transform Infrared–Mass Spectrometry Reactor Cell Setup for Heterogeneous Catalysis with Glovebox Transfer Process to Surface-Chemical Characterization. Rev. Sci. Instrum. 2021, 92, 024105 10.1063/5.0041437. 33648094

[ref39] Saji V. S.; Lee C.-W. Molybdenum, Molybdenum Oxides, and Their Electrochemistry. ChemSusChem 2012, 5, 1146–1161. 10.1002/cssc.201100660. 22693154

[ref40] Bazant M. Z.; Chu K. T.; Bayly B. J. Current-Voltage Relations for Electrochemical Thin Films. SIAM J. Appl. Math. 2005, 65, 1463–1484. 10.1137/040609938.

[ref41] Pavlishchuk V. V.; Addison A. W. Conversion Constants for Redox Potentials Measured versus Different Reference Electrodes in Acetonitrile Solutions at 25 °C. Inorg. Chim. Acta 2000, 298, 97–102. 10.1016/S0020-1693(99)00407-7.

[ref42] Figueiredo M. C.; Ledezma-Yanez I.; Koper M. T. M. In Situ Spectroscopic Study of CO_2_ Electroreduction at Copper Electrodes in Acetonitrile. ACS Catal. 2016, 6, 2382–2392. 10.1021/acscatal.5b02543.

[ref43] Walton J.; Wincott P.; Fairley N.; Carrick A. Peak Fitting with CasaXPS: A Casa Pocket Book; Accolyte Science: Knutsford, UK, 2010. http://www.casaxps.com.

[ref44] Gries W. H. A Universal Predictive Equation for the Inelastic Mean Free Pathlengths of X-Ray Photoelectrons and Auger Electrons. Surf. Interface Anal. 1996, 24, 38–50. 10.1002/(SICI)1096-9918(199601)24:1<38::AID-SIA84>3.0.CO;2-H.

[ref45] Auer A.; Kunze-Liebhäuser J. A Universal Quasi-Reference Electrode for in situ EC-STM. Electrochem. Commun. 2019, 98, 15–18. 10.1016/j.elecom.2018.11.015.

[ref46] Giannozzi P.; Baroni S.; Bonini N.; Calandra M.; Car R.; Cavazzoni C.; Ceresoli D.; Chiarotti G. L.; Cococcioni M.; Dabo I.; Dal Corso A.; De Gironcoli S.; Fabris S.; Fratesi G.; Gebauer R.; Gerstmann U.; Gougoussis C.; Kokalj A.; Lazzeri M.; Martin-Samos L.; Marzari N.; Mauri F.; Mazzarello R.; Paolini S.; Pasquarello A.; Paulatto L.; Sbraccia C.; Scandolo S.; Sclauzero G.; Seitsonen A. P.; Smogunov A.; Umari P.; Wentzcovitch R. M. QUANTUM ESPRESSO: A Modular and Open-Source Software Project for Quantum Simulations of Materials Related Content QUANTUM ESPRESSO: A Modular and Open-Source Software Project for Quantum Simulations of Materials. J. Phys.: Condens. Matter 2009, 21, 395502 10.1088/0953-8984/21/39/395502. 21832390

[ref47] Wellendorff J.; Lundgaard K. T.; Møgelhøj A.; Petzold V.; Landis D. D.; Nørskov J. K.; Bligaard T.; Jacobsen K. W. Density Functionals for Surface Science: Exchange-Correlation Model Development with Bayesian Error Estimation. Phys. Rev. B 2012, 85, 235149 10.1103/PhysRevB.85.235149.

[ref48] Andreussi O.; Hörmann N. G.; Nattino F.; Fisicaro G.; Goedecker S.; Marzari N. Solvent-Aware Interfaces in Continuum Solvation. J. Chem. Theory Comput. 2019, 15, 1996–2009. 10.1021/acs.jctc.8b01174. 30682250

