# Supplementary material for: True Nature of the Transition-Metal Carbide/Liquid Interface Determines Its Reactivity
Source: ACS Catal. 2021 Apr 7;11(8):4920–8. doi: 10.1021/acscatal.1c00415 (PMC8057231; doi:10.1021/acscatal.1c00415)
Supplement: Supplementary file 1 — cs1c00415_si_001.pdf [file cs1c00415_si_001.pdf]

Supporting Information

The True Nature of the Transition Metal Carbide/Liquid Interface Determines its Reactivity

*Christoph Griesser,[†1] Haobo Li,[†2] Eva-Maria Wernig,[†1] Daniel Winkler,[1] Niusha Shakibi Nia,[1] Thomas Mairegger,[1] Thomas Götsch,[1,3,4] Thomas Schachinger,[5] Andreas Steiger-Thirsfeld,[5] Simon Penner,[1] Dominik Wielend,[6] David Egger,[2,7] Christoph Scheurer,[2,7] Karsten Reuter[2,7] and Julia Kunze-Liebhäuser[*1]*

[1]Department of Physical Chemistry, University of Innsbruck, Innrain 52c, Innsbruck, Austria.
[2]Chair of Theoretical Chemistry and Catalysis Research Center, Technische Universität München, 85748 Garching, Germany.
[3]Max Planck Institute for Chemical Energy Conversion, Department of Heterogeneous Reactions, Stiftstraße 34–36, 45470 Mülheim an der Ruhr, Germany.
[4]Fritz-Haber-Institut der Max-Planck-Gesellschaft, Department of Inorganic Chemistry, Faradayweg 4-6, 14195 Berlin, Germany.
[5]University Service Center for Transmission Electron Microscopy, TU Wien, Vienna, Austria.
[6]Linz Institute for Organic Solar Cells (LIOS) / Institute of Physical Chemistry, Johannes Kepler University, Linz, Austria.
[7]Fritz-Haber-Institut der Max-Planck-Gesellschaft, Faradayweg 4-6, Berlin, Germany.

*Corresponding author: Julia.Kunze@uibk.ac.at

Supporting Note 1

Characterization of the Mo_2C film

Atomic force microscopy (AFM), scanning electron microscopy (SEM) of the Mo_2C surface

Atomic force microscopy (AFM) images were recorded in tapping mode on a Dimension 3100 microscope (Veeco) using n-doped silicon probes (force constant 20-80 Nm^{-1}, resonance frequency 256-317 kHz, RTESPW, Veeco). For data processing, Gwyddion[1] was used. Scanning electron microscopy (SEM) measurements were conducted on a Quanta 250 FEG-SEM (ThermoFisher Scientific) at an acceleration voltage of 10 kV. The attached energy dispersive X-ray (EDX) system (Octane Elite 55, EDAX) was operated with TEAM™ software (EDAX) that was used for EDX map generation.

The surface morphology of carburized Mo_2C was investigated by means of AFM and SEM. AFM images reveal that the surface topography is characterized by large terraces of several micrometer width and 10-20 nm height (see Figure S1). According to the calculated root mean square (RMS) roughness, S_q, of 2.75 nm for a (5 × 5) μm^2 and 4.12 nm for a (10 × 10) μm^2 sized surface area, the Mo_2C terrace topography is rather smooth.

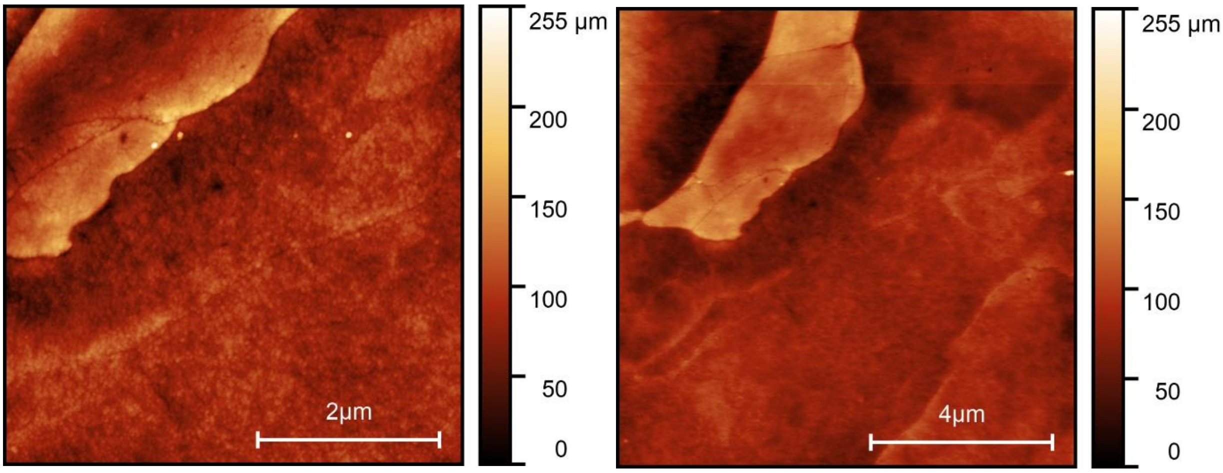

Figure S1. Surface morphology of Mo_2C after its synthesis. AFM images of the carburized Mo_2C surface, with scan areas of (a) (5 × 5) μm^2 and (b) (10 × 10) μm^2.

Energy dispersive X-ray spectroscopy (EDX) was coupled with SEM to obtain SEM-EDX maps of the Mo_2C surface, which confirm its chemical homogeneity (see Figure S2).

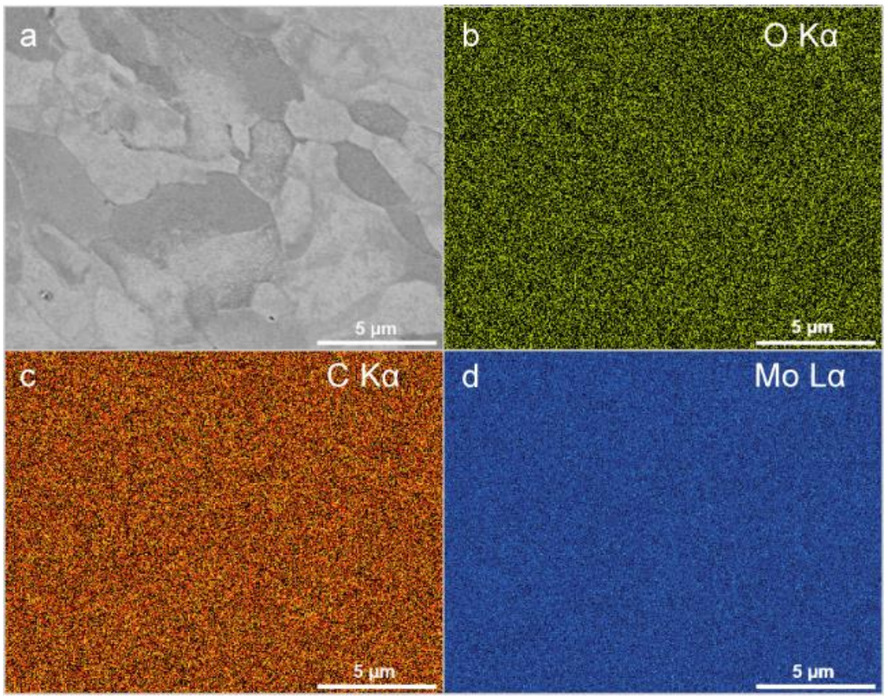

Figure S2. SEM-EDX maps of the carburized Mo_2C film. (a) SEM image (secondary electron contrast) of the mapped surface area; (b)-(d) color-coded elemental maps for the present elements O, C and Mo of representative EDX lines (O K_α, C K_α and Mo L_α).

The SEM image in Figure S2a shows differently oriented crystallographic grains of Mo_2C that constitute the polycrystalline Mo substrate. The EDX maps shown in Figure S2b-d, depict that the elements O, C and Mo are homogeneously distributed over the mapped area. No accumulation of specific elements is observed in the EDX maps. The presence of oxygen (O) can be explained by the fact, that the specimen was exposed to ambient air, which causes surface oxidation.

Transmission electron microscopy (TEM), electron diffraction and EDX imaging of Mo_2C

Transmission electron microscopy (TEM) was used for cross-sectional characterization. A focused ion beam (FIB, Quanta 200 DualBeam, ThermoFisher Scientific) was utilized for the preparation of a thin FIB lamella for TEM analysis. A cross-section was cut across the Mo_2C/Mo interface that was subsequently thinned to about 80 nm using an Ar ion mill. The surface of the Mo_2C film was covered with a protection layer consisting of platinum and carbon (Pt/C). TEM was performed on a Tecnai F20 S-TWIN microscope (ThermoFisher Scientific) operated at an acceleration voltage of 200 kV and equipped with a Gatan Rio camera and an EDAX Apollo XLTW SDD for acquiring EDX spectra, as well as on a ThermoFisher Scientific Talos F200X (200 kV), which was fitted with a Ceta 16M camera and four EDX detectors in a Super-X configuration.

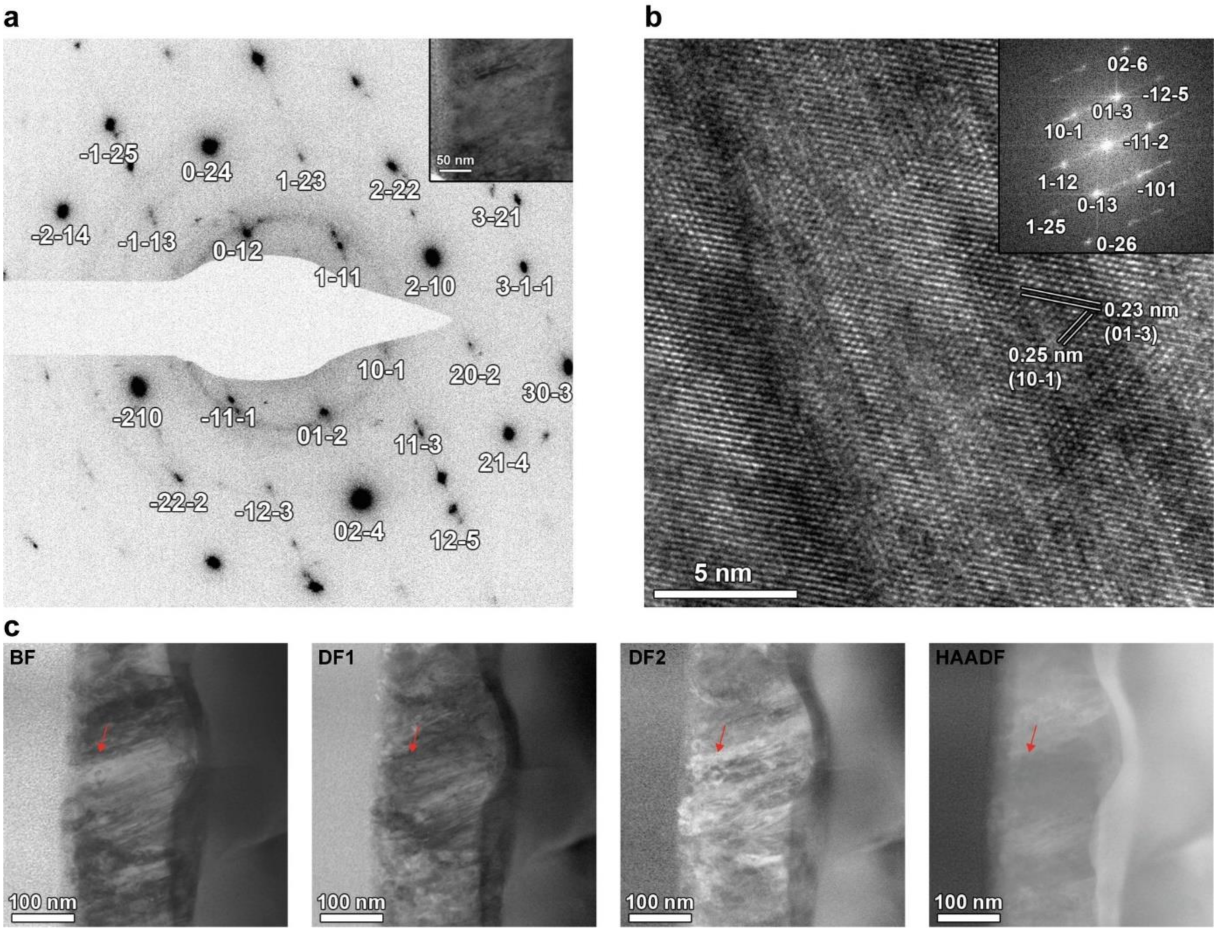

Figure S3. (a) SAED pattern for the area shown in the inset (including a small amount of the Pt protection layer). The scale bar in the image is 50 nm. All indices result from the *P63/mmc* structure.[2] (b) HRTEM image of some Mo_2C crystallites with the corresponding FFT (inset) in a [131] zone axis. (c) STEM images for different scattering angles, ϑ ($\vartheta_{BF} < \vartheta_{DF1} < \vartheta_{DF2} < \vartheta_{HAADF}$), at the same region where the diffraction pattern was acquired. The red arrows mark the same crystallite in each image.

The selected area electron diffraction (SAED) pattern in Figure S3a, acquired in the Mo_2C region, shows a high level of orientation, despite the region at which it was recorded (see the inset) covering multiple crystallites. The almost single-crystalline diffraction pattern suggests a large degree of texturing in the molybdenum carbide phase. The diffraction pattern contains contributions from the Pt/C protection layer (left border of the bright field (BF) image (see Figure S3a, inset). This was deliberately done to avoid any contributions from the large metallic Mo grains that would also yield a spot pattern and make the assignment of lattice planes difficult, whereas the platinum causes easily distinguishable rings. The rings at 0.22 nm, 0.19 nm, 0.14 nm and 0.11 nm show the typical distribution of fcc materials and can be attributed to the (111), (200), (220) and (311) lattice planes of Pt.[3] Molybdenum carbide exists in numerous polymorphs, such as orthorhombic,[4] hexagonal,[2,5] or cubic structures.[6] Despite the differing geometries, the coordination polyhedra and their arrangements are very similar, leading to almost identical diffraction patterns (i.e. all have lattice spacings within the error of most diffraction techniques). However, the angles between the lattice planes differ, which renders it possible to distinguish the structures in the single-crystal-like pattern (see Figure S3a). This was achieved using CrysTBox.[7] Multiple crystal structures were tested using its fitting routines to lead to the best agreement with the reciprocal lattice displayed in Figure S3a. It was found that the data were best fitted by a hexagonal structure (*P63/mmc*) that actually corresponds to a slightly different composition ($Mo_6C_{3.564}$). However, the observed smaller lattice spacings compared to literature values indicate a carbon-deficient structure, i.e. the composition is closer to Mo_2C than to the reference compound of

this crystal structure. The majority of spots, of which some are labelled in Figure S3a (only labelled up to a certain radius to avoid overloading the image), all originate from crystallites oriented on a [121] zone axis. For instance, the {1-11} planes yield spots at approximately 0.245 nm, agreeing well with the theoretical value of 0.256 nm. The {0-12} signals are found at 0.240 nm (expected: 0.245 nm) and the {2-10} planes have a spacing of approximately 0.145 nm, whereas they have 0.150 nm based on reference [2]. As mentioned before, the measured unit cell seems to be smaller than the one found in the literature, which would be in good agreement with a lower carbon content. Despite the good fit of this structure, there are some spots that do not belong to this lattice. These cannot be fitted at the same time as the regular lattice with any of the aforementioned structures because they actually arise due to differently oriented crystallites that were sampled by the diffraction measurement (due to the SA aperture limiting the area only to a diameter of 200 nm). At the top left of the 1-11 spot, there is one at 0.23 nm that can be explained by the (006) planes (0.244 nm) or the (103) planes (featuring a spacing of 0.229 nm). Likewise, between the 11-3 and 12-5 spots, there is an intense reflex at 0.10 nm, which could, for instance, result from the (208) planes at 0.106 nm (although lattice spacings this small are unspecific for a given crystal structure).

The high-resolution TEM (HRTEM) image in Figure S3b can also be described by the same structure, but shows that not all crystallites are oriented the same way. The crystallite shown here is oriented in a [131] zone axis as compared to the [121] axis for the majority of the crystals contributing to the diffraction pattern in Figure S3a (i.e. this particle is slightly rotated around the [100] axis). Some exemplary planes are marked by lines. They include the (01-3) planes, found at 0.23 nm and expected to be at 0.230 nm, as well as the (10-1) planes at 0.25 nm (theoretical: 0.256 nm). The Fast Fourier Transform (FFT) of the $(20 \times 20)\ nm^2$ region (inset in Figure S3b) reveals a good ordering as well, leading to well defined, distinct spots. The different spots in the FFT are labelled and all agree with the calculated [131] zone axis.

The different orientations leading to the overlapping lattices in the diffraction pattern can be seen in Figure S3c, where, scanning transmission electron microscopy (STEM) images recorded for different scattering angles are depicted ($0 \leq \vartheta_{BF} < \vartheta_{DF1} < \vartheta_{DF2} < \vartheta_{HAADF}$). As such, the BF and the two dark field (DF) images primarily contain diffraction contrast, whereas the high-angle annular dark field (HAADF) mainly features Z-contrast. In the BF and DF images, differences in contrast are visible within an image and, for the same crystallite, between images (see for instance the crystallites marked by arrows). This additionally proves that they are differently oriented (otherwise, the contrast would be more uniform as in the molybdenum region on the right-hand sides of the images) and, thus, that the interpretation of the diffraction pattern is plausible. Additionally, the HAADF image reveals that thickness variations within the Mo_2C are only marginal and do not contribute to such strong contrast changes in the other three images.

Since the stoichiometry of the crystallographic reference slightly deviated from the expected composition (that can explain the smaller experimental lattice spacings), the molybdenum-to-carbon ratio was verified by EDX imaging. Figure S4 depicts the integral EDX spectrum of the carbide region of Figure S3c and reveals the composition to be 65 at% Mo and 35 at% C, corresponding well to the nominal stoichiometry of Mo_2C within the uncertainty of the measurement.

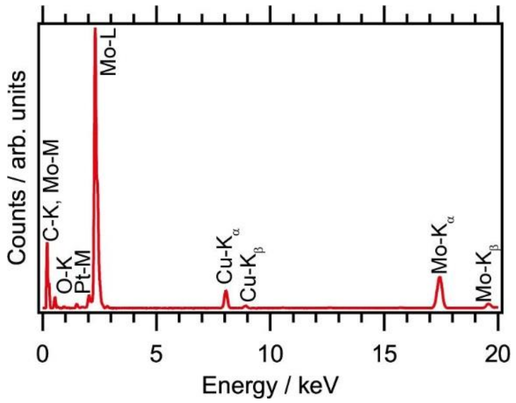

Figure S4. EDX spectrum integrated over the carbide film in Figure S3c showing a composition of Mo_2C, thus corroborating the assignment of the structure.

The thickness used for the absorption correction was estimated to be approximately 80 nm using the low-loss electron energy-loss spectra.[8] There are additional minor signals visible that stem from Pt. This is due to fluorescence from high-energy X-rays emitted by Mo atoms (it is especially visible for the thicker stripe at the interface between the carbide and the metal).

Supporting Note 2

Product analytics

Electrochemistry in aqueous electrolyte

The aqueous electrolytes for cyclo voltammetry and for the XPS studies (see Supporting Note 4) were prepared from concentrated $HClO_4$ (65-71 %, VWR International), NaOH (99.99 %, Sigma-Aldrich) and deionized (DI) water (Milli-Q, 18.2 MΩ, Millipore, Merck). The pH was monitored with a pH meter (827 pH lab, Metrohm) and set to the value of interest, by adjusting the amount of $HClO_4$ added to the solution. All glass, air-tight electrochemical cells were used for the electrochemical measurements. Deaeration of the electrolyte was achieved by purging with Ar (> 99.999 %, Messer) or CO_2 (5.3, 99.9993 %, Linde) for 20 min prior to each measurement. The cyclo voltammetry was conducted in CO_2 (Linde, 5.3) -saturated 0.1 M $KHCO_3$ (SigmaAldrich ≥ 99.95%).

Potential cycling was performed with an an Autolab (Metrohm) potentiostat in hanging meniscus configuration (to make sure that only the Mo_2C surface was exposed to the electrolyte). A flame-annealed carbon rod (Ultra Carbon Corporation) and a saturated calomel or a saturated Ag/AgCl/KCl electrode (SCE, SI Analytics, Schott Instruments) were used as counter (CE) and reference electrode (RE).

Differential electrochemical mass spectrometry (DEMS) and gas chromatography (GC)

For online product identification, a differential electrochemical mass spectrometer (DEMS) (HPR-40, Hiden Analytical, U.K.) equipped with a commercial flow cell (Type A, Hiden Analytical, U.K.) was used. Electron ionization was carried out with an electron energy of 70 eV and an emission current of 500 µA. For detection, a secondary electron multiplier with an acceleration voltage of 3 V was used at an operation voltage of 930 V. A flame annealed carbon rod served as CE and a saturated calomel electrode (SCE) as RE. An Autolab (PGSTAT 302N, Metrohm, Switzerland) with Nova 2.1 operating software was used as potentiostat. As separator between the electrolyte and high vacuum served a PTFE membrane (0.2 µm pore size, 35 µm thickness, Cobetter, China) supported on a stainless-steel

frit. The Mo_2C working electrode (WE) was immersed under potential control at -0.2 V versus the reversible hydrogen electrode (RHE). Linear sweep voltammetry (LSV) between -0.2 and -0.9 V_{RHE} with a scan rate of 2 mV/s was used to determine possible products of the CO_2 reduction. The signals for hydrogen (m/z=2), methane (m/z=15) and ethylene (m/z=26) during the CO_2 electroreduction were recorded simultaneously. A reference measurement was conducted using a polycrystalline Cu-sample as WE in the same potential range with a scan rate of 2 mV/s collecting the same m/z signals.

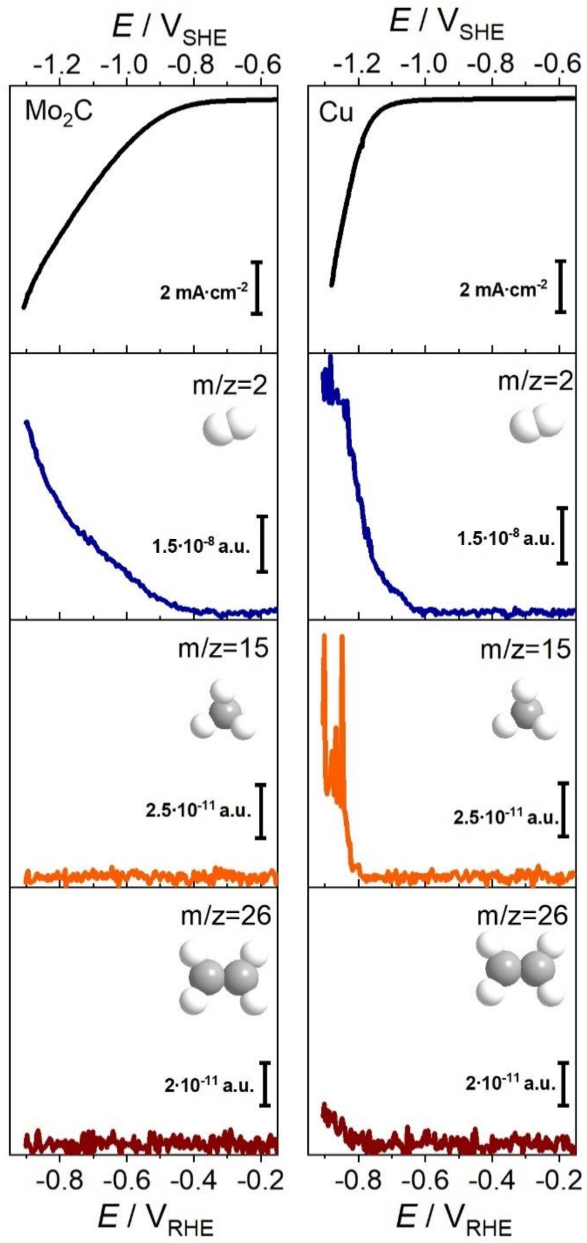

Figure S5. Linear sweep voltammograms of the CO_2 electroreduction at Mo_2C (left) and polycrystalline Cu (right) in 0.1 M $KHCO_3$ (pH 6.9), and the corresponding mass spectrometric linear sweep voltammograms (MSLSVs) for *m/z* = 2 (H_2), 15 (CH_4) and 26 (C_2H_2). Scan rate: 2 mV/s.

Gas chromatography (GC) at pH 3.7

A standard three-electrode cell setup was used for gas chromatography (GC) measurements, with a saturated Ag/AgCl RE (SI Analytics, Schott Instruments) and a carbon rod CE. For the qualitative detection of gaseous products at pH 3.7, the gas chromatograph (Thermo Scientific) was equipped with a thermal conductivity detector (TCD). After 30 min of polarization of the electrode at a selected cathodic potential, a sample volume of 2 mL was extracted from the headspace of the glass cell (250 mL) with a gas-tight syringe and injected in the gas chromatograph, where nitrogen was used as carrier gas. The gas phase was sampled for WE potentials of -0.20, -0.30, -0.40, -0.60 and -0.80 V_{SHE}. Exclusively hydrogen was detected in the GC.

Supporting Note 3

Ab initio thermodynamic calculations

Within the *ab initio* thermodynamics approach, the surface free energy γ is generally defined as[9]

$$\gamma(U,pH) = \frac{1}{2A}\left[G_{\mathrm{surf}}\left(\mathrm{Mo}_x\mathrm{O}_y\mathrm{C}_z\mathrm{H}_w\right) - x\mu_{\mathrm{Mo}} - y\mu_{\mathrm{O}} - z\mu_{\mathrm{C}} - w\mu_{\mathrm{H}}\right] \quad . \qquad (1)$$

Here $G_{\mathrm{surf}}\left(\mathrm{Mo}_x\mathrm{O}_y\mathrm{C}_z\mathrm{H}_w\right)$ is the Gibbs free energy of the surface candidate structure, described via a symmetric slab model with two equivalent surfaces of surface unit-cell area *A* and containing *x* Mo atoms, *y* O atoms, *z* C atoms and *w* H atoms. μ_{species} are the chemical potentials of the respective species. These chemical potentials are defined by various reservoirs with which the surface system is in equilibrium:

- $\mu_{\mathrm{Mo}} = \frac{1}{2}G_{\mathrm{bulk}}(\mathrm{Mo_2C}) - \frac{1}{2}\mu_{\mathrm{C}}$, i.e. the chemical potential of the Mo atoms is defined by the Mo_2C bulk in its hexagonal phase.
- $\mu_{\mathrm{O}} = \mu_{\mathrm{H_2O}} - 2\mu_{\mathrm{H}}$, i.e. the chemical potential of the O atoms is defined by the aqueous environment.
- $\mu_{\mathrm{C}} = \mu_{\mathrm{CO_2}} - 2\mu_{\mathrm{O}}$, i.e. the chemical potential of the C atoms is defined by CO_2 dissolved in the electrolyte.

Rewriting eq. (1), leads to

$$\gamma(U,pH) = \frac{1}{2A}\left[\begin{array}{c} G_{\mathrm{surf}}\left(\mathrm{Mo}_x\mathrm{O}_y\mathrm{C}_z\mathrm{H}_w\right) - \frac{x}{2}G_{\mathrm{bulk}}(\mathrm{Mo_2C}) - \left(z - \frac{x}{2}\right)\mu_{\mathrm{CO_2}} - \\ (y - 2z + x)\mu_{\mathrm{H_2O}} - (w - 2y + 4z - 2x)\mu_{\mathrm{H}} \end{array}\right] \qquad (2)$$

In the difference in eq. (2), the Gibbs free energies of the two solid reservoirs are approximated by their respective total energies,[9] i.e.

$$G_{\mathrm{surf}}\left(\mathrm{Mo}_x\mathrm{O}_y\mathrm{C}_z\mathrm{H}_w\right) \approx E_{\mathrm{surf}}\left(\mathrm{Mo}_x\mathrm{O}_y\mathrm{C}_z\mathrm{H}_w\right) \qquad (3)$$

$$G_{\mathrm{bulk}}(\mathrm{Mo_2C}) \approx E_{\mathrm{bulk}}(\mathrm{Mo_2C}) \qquad (4)$$

The Gibbs free energies of the two liquid reservoirs are calculated as

$$\mu_{\mathrm{CO_2}} = E_{\mathrm{CO_2(molecule)}} + F_{\mathrm{CO_2}}^{\mathrm{vib}} + k_{\mathrm{B}}Tln(c), \qquad (5)$$

where $E_{CO_2(\text{molecule})}$ is the total energy of an isolated CO_2 molecule, $F^{\text{vib}}_{CO_2} = ZPE + \int C_p dT - T\Delta S$ is the vibration correction, and $k_B T ln(c)$ is the concentration correction (with k_B the Boltzmann constant and *T* the temperature). The vibration correction is calculated via density-functional theory (DFT), cf. Table S1 and the description of the computational settings below, and the solubility of CO_2 in water at 100 kPa and 298 K is $c = 6.14 \times 10^{-4}$ mol fraction[10–12]. Similarly,

$$\mu_{H_2O} = E_{H_2O(\text{molecule})} + F^{\text{vib}}_{H_2O} + \Delta\mu_{\text{liquid-gas}}, \quad (6)$$

where the vibrational correction is again provided in Table S1, and $\Delta\mu_{\text{liquid-gas}}$= -0.09 eV[13] is the Gibbs free energy difference between water in the liquid state and gas phase at standard conditions.

Table S1. DFT calculated vibrational corrections in eV at *T* = 298 K.

Species	ZPE	∫CvdT	TΔS[14]
H_2	0.28	0.07	0.40
H_2O	0.71	0.01	0.58
CO_2	0.35	0.01	0.66

This leaves μ_H as the last reservoir and the one that is explicitly dependent on *pH* and the applied bias *U*. Here we employ the computational hydrogen electrode approach[15] and write

$$\mu_H = \frac{1}{2} E_{H_2(\text{molecule})} + F^{\text{vib}}_{H_2} + eU_{\text{SHE}} - k_B T log_{10}(pH). \quad (7)$$

Here, U_{SHE} is the potential referenced to the standard hydrogen electrode (SHE) and the vibrational correction is again provided in Table S1.

In order to model the oxidized surface, the standard 7-layer (1x1)-Mo_2C(110) slab was capped on each side with 1 or 2 surface layers of (100)-oriented MoO_2 in a (1x2) surface unit-cell. This particular MoO_2 orientation and choice of coincidence surface unit-cell leads only to a small lattice mismatch ((2.4% x 2.0%) for phase 1 and (5.1% x 4.3%) for phase 2 of Mo_2C) and a corresponding minimum strain on the oxide overlayer, while simultaneously ensuring a computationally tractable system size.

All three (1x1) terminations possible for Mo_2C(110) were tested: Mo-rich, stoichiometric, and C-rich. For the oxidized systems, also the termination of the (1 or 2 layer) oxide film at the oxide-carbide interface can vary. Here, we tested the three possibilities: Mo-termination, half ML O-termination and full ML O-termination. This combination leads already to 21 test configurations. In addition, either the surface of the carbide or of the oxide film can be terminated with varying amounts of O or hydroxyls: Here, we tested five different O or OH coverages: 0 ML, ¼ ML, ½ ML, ¾ ML and 1 ML. In total, this thus gives rise to 315 considered test structures for each Mo bulk carbide phase.

Free energy calculations

Adsorption free energies were calculated as G_{ad}(*COOH) = $E_{*COOH/surface}$ - $E_{surface}$ - μ_{CO_2} - μ_H + ΔG_{corr}; G_{ad}(*H) = $E_{*H/surface}$ - $E_{surface}$ - μ_H + ΔG_{corr}, where $E_{*COOH/surface}$, $E_{*H/surface}$, $E_{surface}$ are the total energies of structure ⑧ in Fig. 2 with adsorbed *COOH, *H and without adsorbate, respectively. ΔG_{corr} contains the vibrational free energy correction (ZPE + $\int C_p dT$ - *TS*, zero-point energy ZPE, enthalpic temperature correction $\int C_p dT$, and entropic correction *TS*) and the implicit solvation correction (ΔG_{sol}).

Surface Pourbaix diagram of the hexagonal $(\beta) - 1$ Mo_2C phase

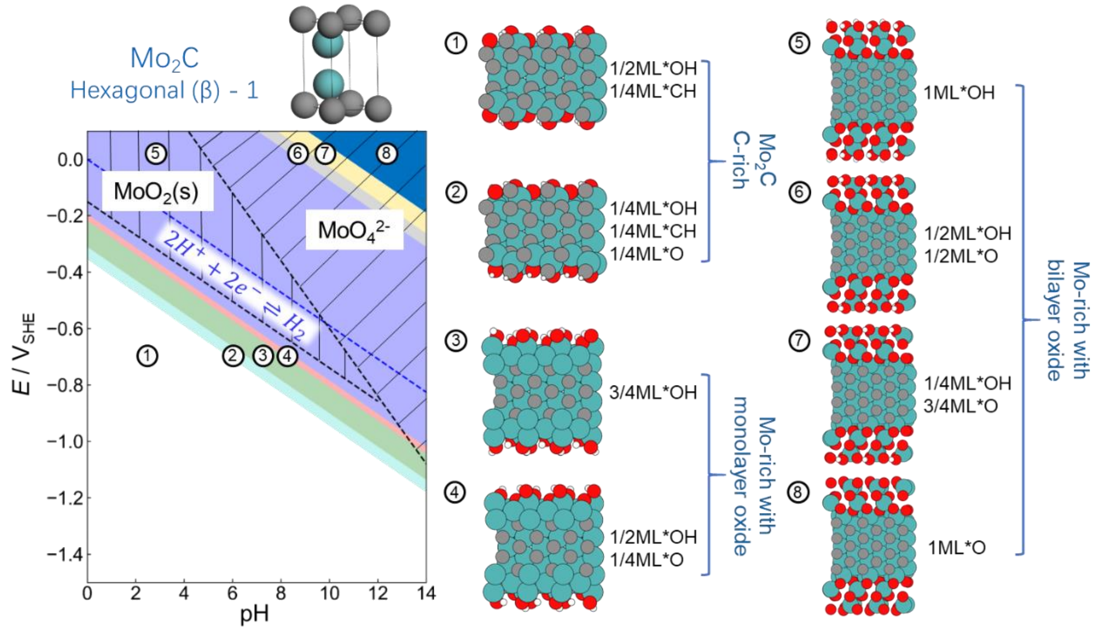

Figure S6. Surface Pourbaix diagram for MoO_2(100)/Mo_2C(110) for the hexagonal phase β-1 of Mo_2C with the composition of Mo-C-O-H. The stability region of bulk MoO_2 and dissolved MoO_4^{2-} as known from the experimental Pourbaix diagram for the parent metal[16] are indicated by black dashed areas. The blue dashed line indicates the onset of the hydrogen evolution reaction (HER). This figure is fully analogous to Figure 2 in the main text for the more stable hexagonal Mo_2C bulk phase. Please refer to the main manuscript text for further details.

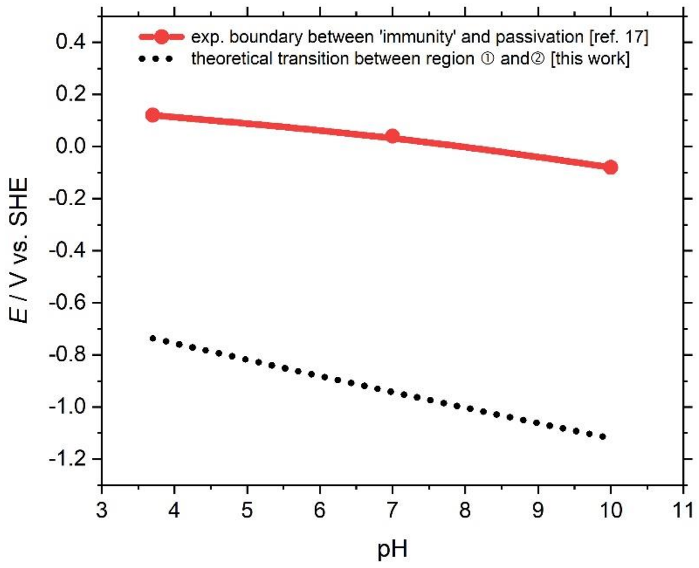

Figure S7. Comparison of the transition from apparent thermodynamic immunity of Mo_2C to a passivated surface, i.e. line between regions ① and ② in Figure 2 (this work, black dots) and from apparently immune Mo_2C to passivation from ref. [17] (red line) at pH 3.7, pH 7 and pH 10.

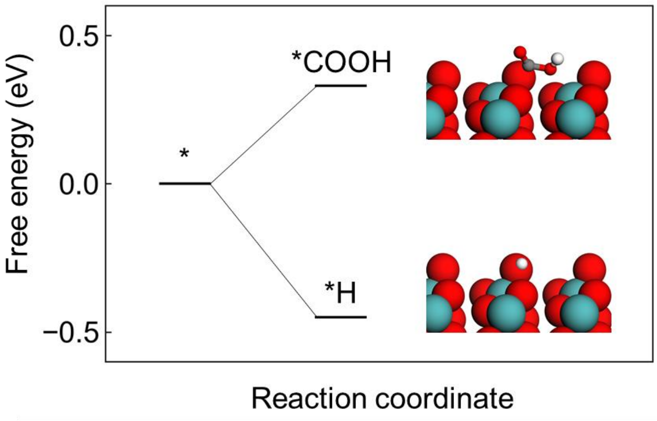

Figure S8. Free energy diagram of the first protonation step of HER and CO_2RR on the MoO_2(100) covered Mo_2C(110) surface, cf. structure ⑧ in Figure 2.

Supporting Note 4

Experimental Pourbaix diagram

Table S2: Experimental scattering of the amount of native oxide on pristine Mo_2C surfaces after air exposure and after quantification of XPS spectra recorded at an angle of 0°.

sample #	elemental composition / %			Mo 3d fit components / %	
	C	Mo	O	$Mo_{carbidic}$	Mo^{6+}
**1**	28.9	39.2	31.9	88.2	11.9
**2**	37.4	40.0	22.7	93.7	6.3
**3**	34.5	42.1	23.4	95.6	4.4
**4**	33.8	38.9	27.3	93.9	6.1
**5**	34.4	39.8	25.9	95.2	4.8
**6**	31.3	47.6	21.1	93.3	6.7
**average**	**33.4**	**41.3**	**25.4**	**93.3**	**6.7**
**standard deviation**	**± 3.1**	**± 1.3**	**± 3.7**	**± 3.0**	**± 3.0**

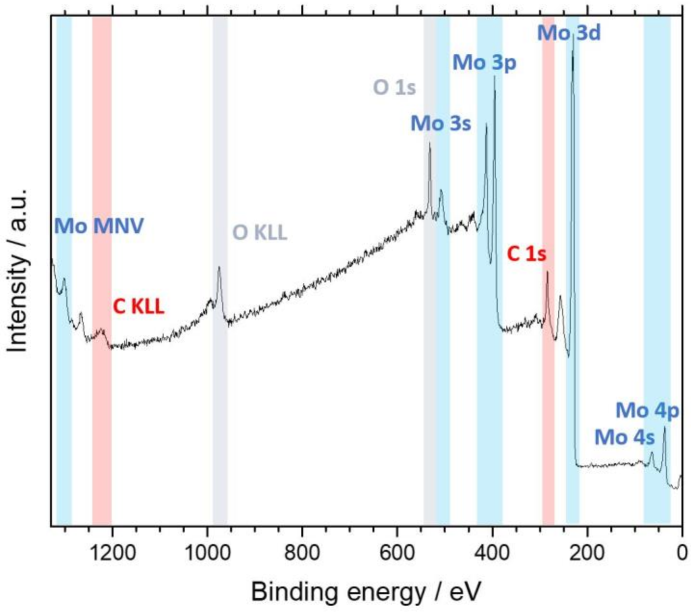

Figure S9. XPS survey scan of the carburized Mo_2C surface, demonstrating the purity of the Mo_2C surface, as no foreign element was detected.

High resolution X-ray photoelectron spectroscopy (XPS) data evaluation

The C 1s region comprises an asymmetric peak at ~283.1 eV that shows the presence of molybdenum carbides (denoted as $C_{carbidic}$ in this paper), which confirms the successful carbide synthesis. The asymmetric peak at ~284.2 eV corresponds to graphitic carbon (denoted as $C_{graphitic}$), whereas the symmetric one at ~284.8 eV matches hydrocarbon polymers (C-C, C-H). Oxygenated carbon species give rise to signals at ~286.5 eV and ~288.0 eV, assigned to C-OH and C-O-C, and to C=O, respectively. The most intense signal in the Mo 3d region is located at ~228.0 eV and originates from the $3d_{5/2}$ spin orbit component of molybdenum carbide, with its $3d_{3/2}$ peak at ~231.15 eV. The oxidation state of Mo in carbides is not completely understood and still debated in the literature. It has been interpreted as metal-like Mo^0 by Chen *et al.*[18] and as Mo^{2+} by Murugappan *et al.*[19]. Therefore, we define carbide related signals in the Mo 3d spectra as $Mo_{carbidic}$ in order to be non-committal concerning its oxidation state. The broadening of the $Mo_{carbidic}$ $3d_{3/2}$ with respect to the $Mo_{carbidic}$ $3d_{5/2}$ peak can be explained by Coster-Kronig transitions, i.e. Coster-Kronig broadening. The Mo^{6+} oxide peaks appear at ~232.30 eV ($3d_{5/2}$) and ~235.4 eV ($3d_{3/2}$). The fit parameters for the high-resolution spectra are given in Table S3.

Table S3. Fit parameters for XPS spectra (C 1s and Mo 3d regions) of the pristine surface after air exposure. Peak positions of the fit components are listed with the corresponding full width at half maximum (FWHM) values given in brackets.

Peak position (FWHM) / eV								
C 1s					Mo $3d_{5/2}$		Mo $3d_{3/2}$	
$C_{carbidic}$	$C_{graphitic}$	C-C C-H	C-OH C-O-C	C=O	$Mo_{carbidic}$	Mo^{6+}	$Mo_{carbidic}$	Mo^{6+}
283.1 (0.8)	284.2 (0.8)	285.1 (1.5)	286.6 (1.5)	288.1 (1.5)	228.0 (0.7)	232.3 (1.5)	231.1 (0.8)	235.4 (1.5)

Angle resolved XPS studies of the electrode

Angle resolved XPS measurements were carried out to gain information of the chemistry and thickness of the electrodes' topmost layers. The surface sensitivity is enhanced through tilting the angle between the surface normal of the specimen with respect to that of the analyser. The dependence of the information depth on the angle θ between sample and analyser follows the description given by equation (8):

$$I(d) = I_0 * e^{\frac{-d}{\lambda * \cos(\theta)}} \quad (8)$$

Hence, the intensity of a signal $I(d)$ depends on the depth distribution of the corresponding species, the inelastic mean free path (λ) and the angle (θ) between sample and analyser. Figure S10 depicts the deconvoluted C 1s and Mo 3d regions of the pristine electrode. In the C 1s spectrum (Figure S10a), the carbide signal at ~283.1 eV dominates at 0°. With increasing angle, the graphitic carbon signal (at ~284.2 eV) intensity and, to a minor degree, the intensity of oxygenated carbons increases, while the carbide signal intensity decreases.

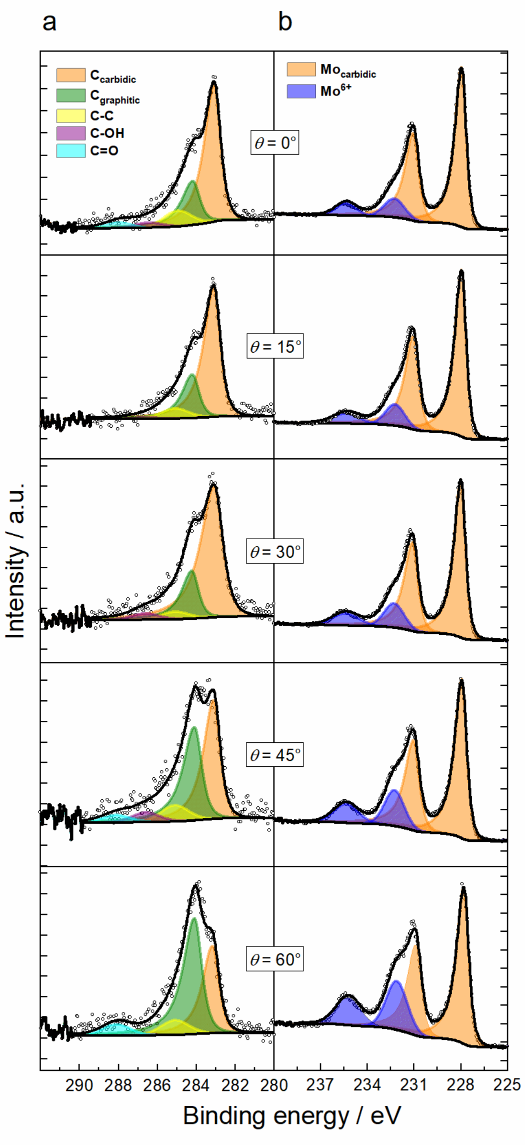

Figure S10. Angle resolved XPS measurements of the Mo_2C electrode. (a) depicts the deconvoluted C 1s region and (b) the deconvoluted Mo region at different values of θ ranging from 0° to 60°.

In the Mo 3d spectrum (see Figure S10b), two different species can be distinguished, Mo_2C (orange) and MoO_3 (blue). Their relative intensities strongly depend on the angle between analyser and sample normals. While the amount of Mo_2C decreases with increasing angle, the MoO_3 signal intensities rise. This strongly suggests a layered structure (MoO_3 on top of the bulk Mo_2C). The thickness of the oxide layer on top of the Mo_2C can be calculated for every angle by utilizing the following equation proposed by Carlson *et al.*[20].

$$d = \lambda_o * \cos(\theta) \ln(\frac{N_c \lambda_c I_o}{N_o \lambda_o I_c} + 1) \quad (9)$$

This equation correlates the oxide layer thickness (d) to the inelastic mean free path (IMFP) of the oxide (λ_o), the angle between sample and analyser normals θ, the IMFP of the carbide (λ_c) and the relative intensities of the oxide (I_o) and the carbide (I_c). The volume densities of the metal atoms in the oxide (N_o) and in the carbide (N_c) also influence the calculated oxide layer thickness. In this work, an IMFP of 18 nm was assumed for both the oxide and the carbide. The atomic volume densities were calculated[20] from the bulk densities, which are 4.69 g cm^{-3} and 8.9 g cm^{-3} for the carbide and the oxide, respectively. The surface oxide thickness determined with equation (9) is shown in Figure S11.

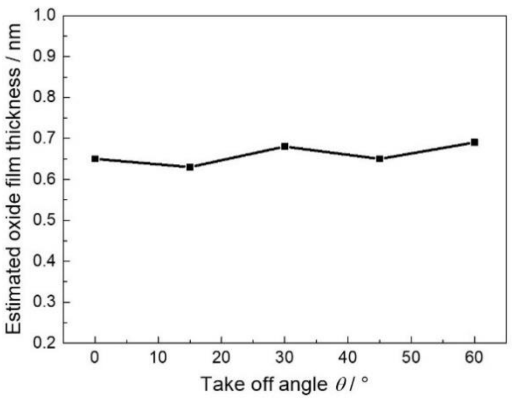

Figure S11. MoO_3 thickness determined with angle resolved XPS and equation (9). The values are calculated from the deconvoluted Mo 3d spectra for each angle separately.

For all measured angles, an oxide layer thickness between 0.62 nm and 0.70 nm is estimated. An average of (0.66 ± 0.02) nm results, which fits nicely to the expected thickness of one double layer of edge-shared MoO_6 octahedra constituting layered MoO_3.[21] The small standard deviation regarding the oxide layer thickness is a measure for the high quality of the Mo 3d fit.

This result is well in line with the XPS sputter depth profile, which is discussed in the following section, as short sputter cleaning is sufficient to remove the surface oxide.

Quantification of EDX line scan and XPS sputter depth profile

To measure the carbide and native oxide thicknesses, XPS sputter depth profiles have been conducted using Ar sputtering in a series of sputter steps ranging from 10 s to 1000 s per step to mill the whole film down to the substrate. The corresponding C 1s, Mo 3d and O 1s high resolution (HR) XPS spectra are depicted as waterfall plots in Figure S12. After a total sputter time of 10,000 s, the carbide film was completely removed from the Mo substrate.

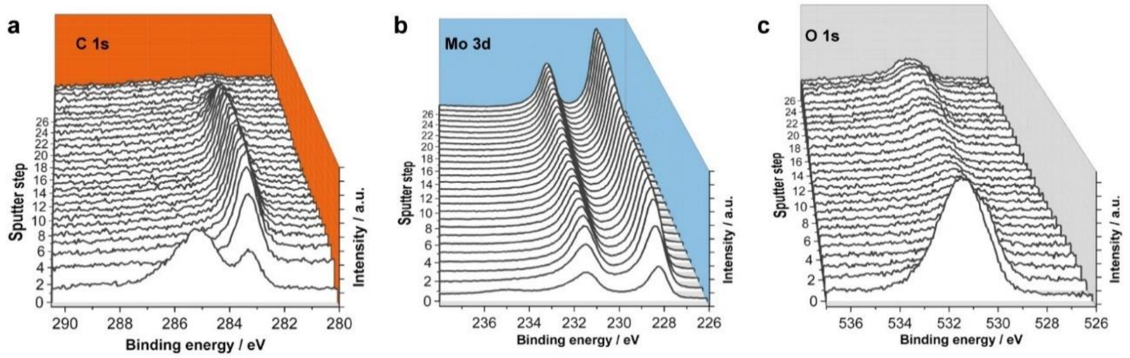

Figure S12. XPS sputter depth profile showing the evolution of the (a) C 1s, (b) Mo 3d and (c) O 1s binding energy (BE) regions over the entire molybdenum carbide film.

The C 1s signal shows that after 60 s of sputtering, all adventitious carbon species are entirely removed from the surface. The surface oxide in the Mo 3d region is also eliminated by this treatment. Residues of oxygen visible in the O 1s region can be attributed to dissolved oxygen in the carbide or metal lattice.

For calibration of the XPS depth profile, the Mo_2C film thickness has been determined via TEM-EDX studies using a FIB lamella of the carburized specimen, where the Mo_2C film is embedded between the polycrystalline Mo substrate and a Pt/C protection layer, over which an EDX line scan was measured. The EDX spectra were processed with HyperSpy Python software[22] for signal deconvolution in the low energy range between 0.0 keV and 0.7 keV. This is necessary to avoid artefacts arising from differing self-absorption properties of the low-energy C-K_α and O-K_α lines and the relatively high-energy Mo-L or Mo-K lines. Four characteristic EDX lines are found in the selected spectral region corresponding to the Cu-M (originating from the grid used to mount the FIB lamella), Mo-M, C-K_α and O-K_α lines, located at 0.09 keV, 0.19 keV, 0.28 keV and 0.52 keV, respectively. Signals were fitted using a 6th order polynomial background (see Figure S13).

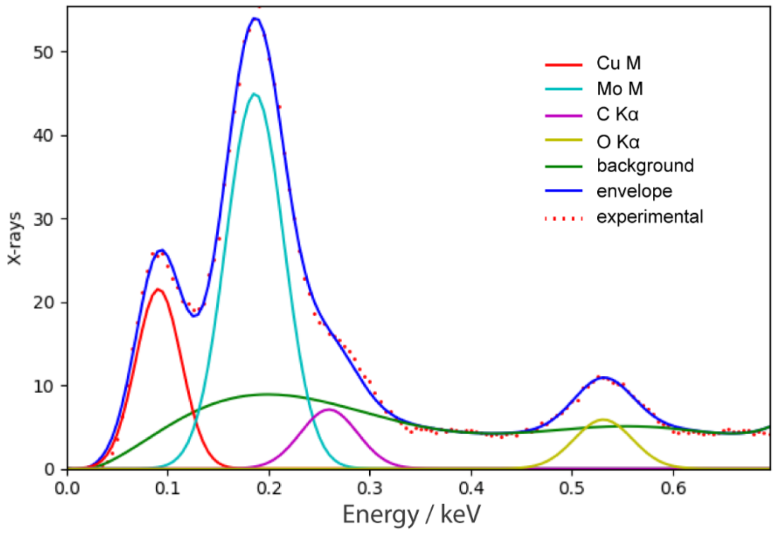

Figure S13. EDX fit of a selected spot from the line scan recorded on the carburized film, processed with HyperSpy.

The EDX peak areas used for the depth profile allow for determination of the film thickness and qualitative analysis. Quantification was not meaningful because of unknown *k* factors. The qualitative

cross-section (see Figure S14a) shows the homogeneity of the film through nearly constant apparent concentrations of Mo and C. The intensity of the O-K_α line is not as homogeneous, which might result from fluorescence from the high-energy Mo lines or self-absorption. A sigmoidal fit of the C-K_α line at both interfaces (protective Pt/C layer/carbide and carbide/substrate) was used to estimate the film thickness to 230 nm. Figure S13b shows a high angle annular dark field (HAADF) image of the FIB lamella used to determine the cross-section. The arrow in the HAADF image indicates the position of the cross-section shown in panel a.

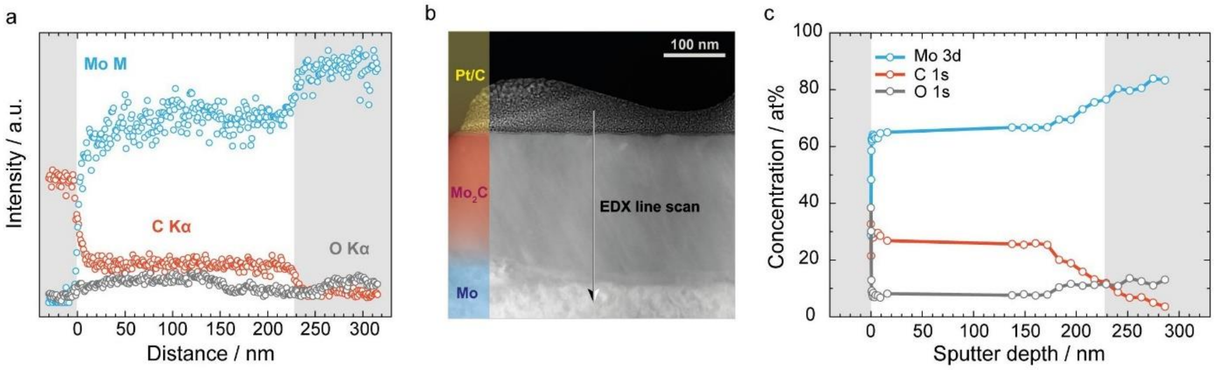

Figure S14. Cross-sectional chemical composition of the Mo_2C film. (a) EDX line scan over the cross-section for determination of the film thickness; (b) HAADF image of the Mo/Mo_2C interface in the FIB lamella; (c) calibrated XPS sputter depth profile.

The resulting quantitative evaluation of the XPS sputter depth profile (Figure S14c) shows a continuously homogeneous distribution of Mo, C and O across the film, after removal of oxide and adventitious carbon from the surface. A film thickness of 230 nm is used for calibration of the sputter depth. Average values for the relative amounts of Mo, C and O in the film are (66.1 ± 1.0) at%, (26.3 ± 1.2) at% and (7.7 ± 0.5) at%, respectively. If a stoichiometry of Mo_2C is considered, the excess 13.5 at% Mo can be bound to the small amount of O, which is omnipresent in the bulk film and which might occupy interstitial lattice sites. Oxygen can dissolve in the metal substrate, which could be the reason for the increase of the O concentration inside the Mo metal substrate. Preferential sputtering is not considered to take place, because both the XPS depth profile and the EDX cross-section (from the FIB lamella) show the same trend.

XPS after synthesis without exposure to air

After synthesis of Mo_2C in a vacuum-assisted annealing station, and transfer to the XPS chamber under protection from ambient air, the resulting pristine surface does not exhibit Mo oxide species, which is shown in Figure 3 (middle panel) in the main paper. However, exposure to air for 15 minutes already results in a significant increase in the oxide signal, as well as in the Mo:$C_{carbidic}$ ratio (Table S4). This clearly shows the high susceptibility of the surface to oxidation upon short exposure to the ambient air. Intriguingly, cathodic polarization of the electrode at -0.2 V_{SHE} in oxygen free $NaClO_4$ (0.1 M, pH 3.7) causes an even higher increase in the oxygen signal, and the Mo:C ratio (Table S4), exclusively due to MoO_2 formation, with an estimated MoO_2 thickness of 0.6 – 0.7 nm.

Table S4. Quantification of the XPS spectra measured at 0° take-off angle, shown in Figure 3 in the main paper and Supporting Figure 14, emphasizing the susceptibility of the Mo_2C surface to oxidation.

Sample	Atomic fraction / at.%					Stoichiometric ratio
	C	Mo	O	$C_{carbidic}$	Mo	Mo:C
**pristine**	47.1	44.7	8.2	22.2	44.7	**2.01**
**15 min air exposure**	37.4	45.3	17.4	20.6	45.3	**2.20**
**300 min air exposure**	36.1	40.0	23.9	16.6	40.0	**2.41**
**15 min polarization**	61.0	20.5	18.6	8.0	20.5	**2.56**

Native oxide is present after very short times of air exposure (see Figure S15).

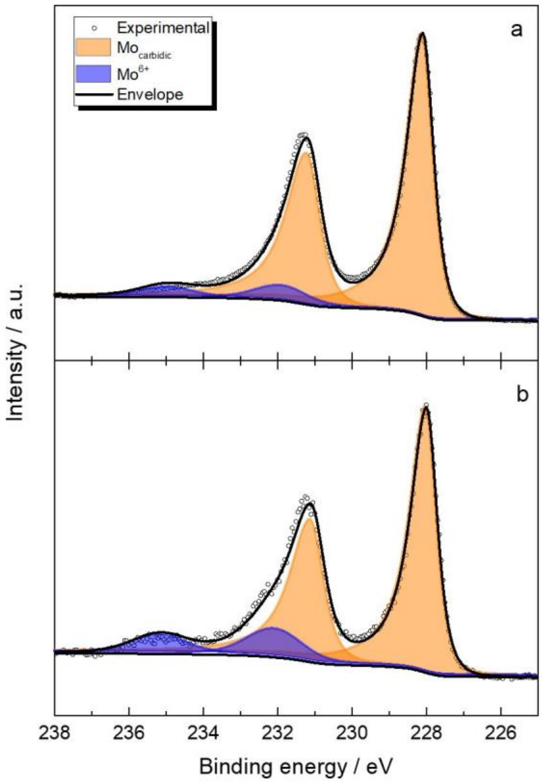

Figure S15. XPS spectra of the Mo 3d region of a freshly synthesized Mo_2C surface (a) after 15 min, and (b) after 300 min air exposure. Escape angle: 0°.

Hence, the surface coverage of Mo_2C electrodes with oxides after immersion into an electrolyte solution seems to be independent of the surface chemistry before polarization, i.e. whether the sample was exposed to air prior to its exposure to the electrolyte solution or not, as the XPS spectra afterwards both exhibit a very similar behavior.

Table S5. Fit parameters for XPS spectra (C 1s and Mo 3d regions) of the surface after 15 min polarization at -0.2 V_{SHE} in $NaClO_4$ (pH 3.7) shown in Figure 3 (bottom panel) in the main paper. Peak positions of the fit components are listed with the corresponding full width at half maximum (FWHM) values given in brackets.

Peak position (FWHM) / eV								
C 1s					Mo $3d_{5/2}$		Mo $3d_{3/2}$	
$C_{carbidic}$	C-C<br>C-H	C-OH<br>C-O-C	C=O	O-C=O	$Mo_{carbidic}$	Mo^{4+}	$Mo_{carbidic}$	Mo^{4+}
283.1<br>(0.7)	285.1<br>(1.6)	286.6<br>(1.6)	288.1<br>(1.6)	289.6<br>(1.6)	228.0<br>(0.7)	230.3<br>(1.8)	231.1<br>(0.8)	233.4<br>(2.2)

Quasi in-situ XPS studies

For the transport of the electrodes from the glovebox to the XPS analyzing chamber without exposure to the ambient, a home-built transfer cell (Figure S16) was utilized.

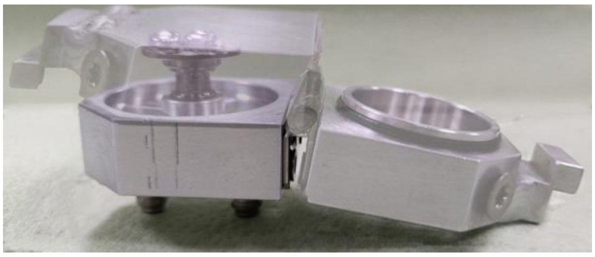

Figure S16. Image of the home-built transfer cell for the transport of the electrodes without exposure to air.

To define the potentials of interest for the quasi *in-situ* studies,[23] cyclic voltammograms (CVs) were recorded in hanging meniscus configuration in 0.1 M $NaClO_4$ at three different pH values (Figure S17). The WEs were immersed at reductive potentials, where no net anodic currents are detectable, being -0.2 V_{SHE} (pH 3.7), -0.3 V_{SHE} (pH 7), or -0.4 V_{SHE} (pH 10). CVs were recorded between the respective immersion potential and -0.8 V_{SHE} or -1.6 V_{SHE} at a scan rate of 20 mVs^{-1}. In the CV recorded at pH 3.7 (black), three different regions are visible. Between -0.2 V_{SHE} and -0.35 V_{SHE}, charging of the double layer occurs. This is followed by the appearance of a cathodic peak with an onset at ~ -0.40 V_{SHE}, which is related to proton reduction[24], the high reduction current found at more negative potentials is related to reduction of neutral water. Therefore, the effect of cathodic polarization on the surface composition after applying -0.2 V_{SHE} (only charging of the double layer), -0.4 V_{SHE} (proton reduction peak), -0.6 V_{SHE} (onset of the water reduction) and -0.8 V_{SHE} (far in the water reduction region) was investigated at pH 3.7. At pH 7, where the CO_2 reduction experiments are conducted, the potentials were lowered down to very negative values of -1.6 V_{SHE} where high cathodic currents are detected.

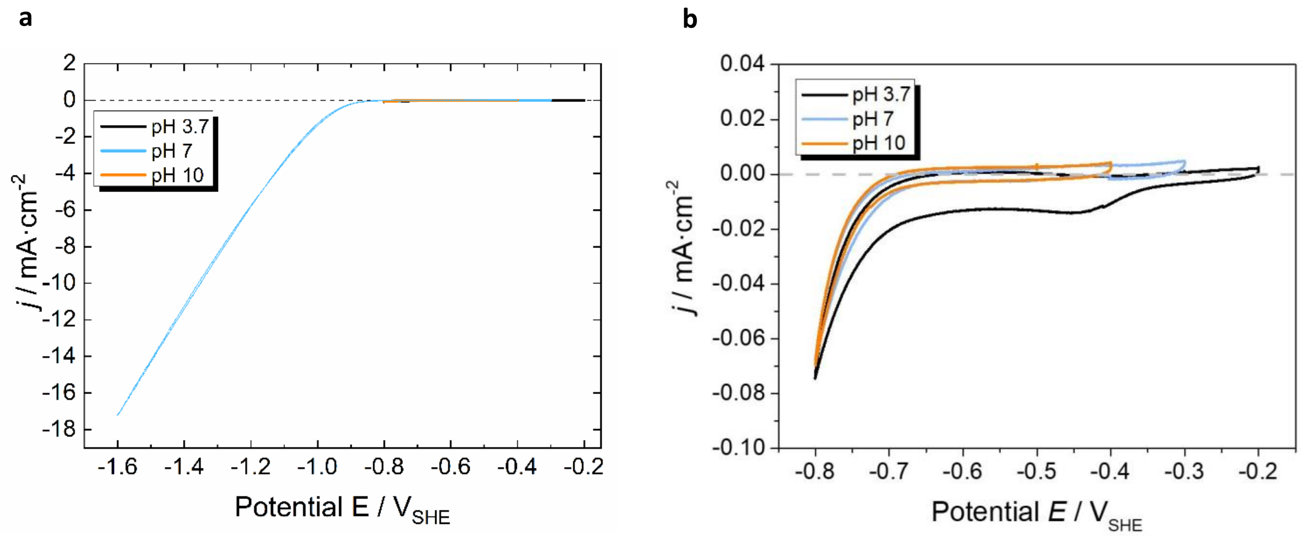

Figure S17. Cyclic voltammograms (CVs) of the Mo_2C electrode in 0.1 M $NaClO_4$ at pH 3.7 (black), pH 7 (blue) and pH 10 (orange). Measurements were conducted with a scan rate of 20 mV s^{-1} in Ar saturated electrolyte in hanging meniscus configuration. A zoom is shown in panel b.

The separation of the proton and water reduction is not found at pH 7 and pH 10, because the proton concentrations are too low at these high pH values. The water reduction occurs approximately at the same potential and at -0.8 V_{SHE}, no significant difference in the current densities can be observed for the three different electrolytes. The potentials of interest for pH 7 and pH 10 were shifted by -0.1 V and -0.2 V with respect to the potentials chosen at pH 3.7. Due to the high amount of hydrogen emerging at -0.9 V_{SHE} and -1.0 V_{SHE} these potentials were not used for *ex-situ* emersion XPS studies, because no reliable results can be expected at these potentials.

Determination of the experimental Pourbaix diagram

The XPS spectra containing the data used in the experimental Pourbaix diagram were recorded at an angle of 60° between sample and analyzer normal to have improved surface sensitivity. The HR spectra of the C 1s and Mo 3d regions are shown in Figure S18a-c.

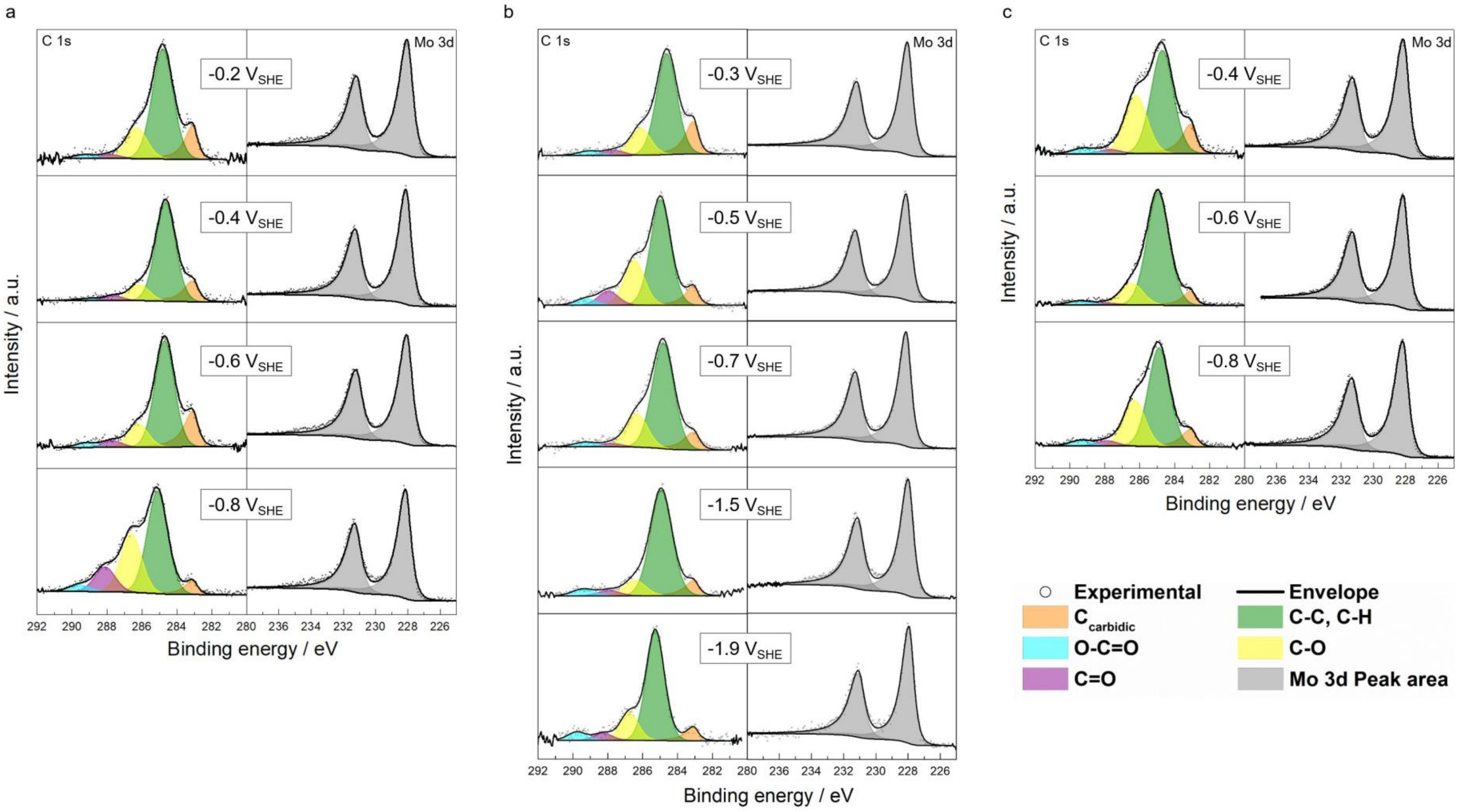

Figure S18. Deconvoluted XPS spectra of the C 1s region (left panels) and the Mo 3d region (right panels) of Mo_2C after emersion from solutions of (a) pH 3.7, (b) pH 7 and (c) pH 10. The spectra were acquired after polarization of the electrode in Ar saturated 0.1 M $NaClO_4$ for 15 min at different potentials vs. SHE (see insets). All spectra were recorded at an angle of 60° between the surface normals of the samples and the analyzer.

After polarization of the electrodes at any chosen potential, no Mo^{6+} signals have been observed anymore (Figure S18a-c, right panels), and it was not possible to reliably distinguish between the Mo^{4+} and $Mo_{carbidic}$ species via deconvolution of the Mo 3d spectra. Therefore, another option was utilized, which is based on the calculation of the Mo:$C_{carbidic}$ ratio. In UHV studies, this method was already used to determine if overlayers are formed on Mo_2C single crystals by St. Clair *et al.*[25]. The Mo:$C_{carbidic}$ ratio enables the calculation of the oxide layer thickness on top of the Mo_2C electrode. $C_{carbidic}$ is determined through deconvolution of the C 1s signals (Figure S18a-c, left panels). A Mo:$C_{carbidic}$ ratio of 2 is attributed to pure Mo_2C, whereas the measured higher ratio indicates the presence of additional oxides. The ratio > 2 agrees well with the O 1s spectra (see Figure S12c), in which a broad peak between 529 eV and 535 eV is observed. This peak can be attributed to oxides and hydroxides (from the surface film) as well as to oxygenated carbon species (related to the adventitious carbon contamination).

The relation between the Mo:$C_{carbidic}$ ratio and estimated oxide thickness is depicted in Figure S19. For the calculation of this relationship it was assumed that the oxide overlayer consists of MoO_2 so that equation (7) can be applied. Possibly present suboxides in the surface film are not considered for this assumption. A change in the overall trend of the experimental Pourbaix diagram is however excluded, because the simplification was equally applied to all E/pH values. The density of MoO_2 (6.47 g cm^{-3}) was used (instead of the density of MoO_3), the other parameters employed in equation (7) were identical to those applied for the determination of the native oxide layer thickness.

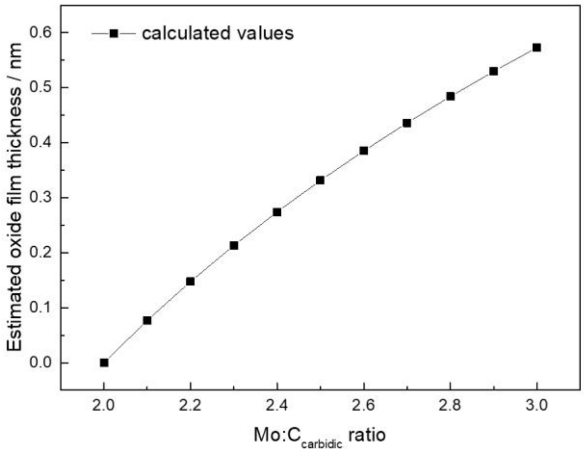

Figure S19. Correlation between the estimated oxide film thickness and the Mo:$C_{carbidic}$ ratio.

A detailed information on the fit parameters is given in Table S6. A Gaussian-Lorentzian GL(30) peak shape was utilized for all signals except for the Mo_2C peaks, where asymmetric shapes were used. The binding energies as well as the FWHM values of the C-O, C=O and O-C=O species were constrained with respect to the C-C/C-H signal. The position of the $C_{carbidic}$ signal was used for charge correction (set at 283.1 eV). Its FWHM was strictly constrained to 0.7 - 0.8 eV, as this signal is the most important one regarding the data evaluation. In the Mo 3d region no constraints were utilized, but the spin orbit splitting, where the Mo $3d_{3/2}$ was set to 3.15 eV above the Mo $3d_{5/2}$ component, was strictly considered.

Table S6. Fit parameters for the deconvolution of the C 1s region. The fits relate to Figure S18. The Mo 3d peak was integrated over the whole region, as a reliable deconvolution was not possible.

Sample		Binding energy [FWHM] / eV						
pH	Potential / V_{SHE}	Carbide	C-C, C-H	C-OH, C-O-C	C=O	O-C=O	$Mo_{5/2}$	$Mo_{3/2}$
3.7	-0.2	283.1 [0.70]	284.8 [1.41]	286.3 [1.41]	287.8 [1.4]	289.3 [1.41]	228.0 [0.83]	231.2 [0.92]
	-0.4	283.1 [0.79]	284.6 [1.33]	286.1 [1.33]	287.6 [1.33]	289.1 [1.33]	228.1 [0.78]	231.3 [0.90]
	-0.6	283.1 [0.77]	284.7 [1.31]	286.2 [1.31]	287.2 [1.31]	289.2 [1.31]	228.1 [0.81]	231.2 [0.91]
	-0.8	283.1 [0.70]	285.1 [1.39]	286.6 [1.39]	288.1 [1.39]	289.6 [1.39]	228.1 [0.80]	231.2 [0.89]
7	-0.3	283.1 [0.71]	284.6 [1.37]	286.1 [1.37]	287.6 [1.37]	289.1 [1.37]	228.0 [0.76]	231.2 [0.89]
	-0.5	283.1 [0.72]	285.0 [1.34]	286.5 [1.34]	288.0 [1.34]	289.3 [1.34]	228.1 [0.76]	231.3 [0.87]
	-0.7	283.1 [0.85]	284.8 [1.45]	286.3 [1.45]	287.8 [1.45]	289.3 [1.45]	228.1 [0.76]	231.3 [0.88]
	-1.5	283.1 [0.75]	284.9 [1.45]	286.4 [1.45]	287.9 [1.45]	289.4 [1.45]	228.0 [0.78]	231.1 [0.90]}
	-1.9	283.1 [0.7]	285.2 [1.22]	286.7 [1.22]	288.2 [1.22]	289.7 [1.22]	227.9 [0.72]	231.1 [0.89]
10	-0.4	283.1 [0.76]	284.7 [1.50]	286.2 [1.50]	287.7 [1.50]	289.2 [1.50]	228.2 [0.84]	231.3 [0.95]
	-0.6	283.1 [0.70]	284.9 [1.50]	286.4 [1.50]	287.9 [1.50]	289.4 [1.50]	228.2 [0.79]	231.3 [0.90]
	-0.8	283.1 [0.80]	284.9 [1.44]	286.3 [1.44]	287.9 [1.44]	289.4 [1.44]	228.2 [0.84]	231.3 [0.95]
Constraints	Peakshape	LA(1.1,2.3,2)	GL(30)	GL(30)	GL(30)	GL(30)	LA(1.1,2.3,2)	LA(1.1,2.3,2)
	Assymtry index	0.3515	0	0	0	0	0.3515	0.3515
	Binding Energy	283.1	arbitrary	[C-C] + 1.5	[C-C] + 3	[C-C] + 4.5	arbitratry	$Mo_{5/2}$ + 3.15
	FWHM	0.7 -0.85	1.22-1.5	[C-C] * 1	[C-C] * 1	[C-C] * 1	arbitratry	arbitratry

Atomic fractions were calculated from the XPS spectra and are shown in Table S7. This and the quantification of the corresponding C 1s spectra (Table S8) were utilized to calculate the Mo:$C_{carbidic}$ ratios and the related oxide layer thicknesses. The intervals given in Table S8 represent the error bars shown in Figure 4 in the main paper. They result from the small changes occurring through the fit procedure of the C 1s spectra that leads to viable fit parameters. The final values given in Table S8 correspond to the most reliable fits.

Table S7. Quantification of the XPS spectra of the Mo_2C electrodes recorded at a 60° take-off angle after polarization in 0.1 M $NaClO_4$ at different pH values for the determination of the overall atomic fraction.

Sample		Atomic fraction / at.%		
pH	*E* / V_{SHE}	C	Mo	O
3.7	-0.2	49.8	20.9	29.4
	-0.4	53.7	19.7	26.6
	-0.6	53.9	23.4	22.7
	-0.8	69.6	8.9	21.5
7	-0.3	57.9	25.0	17.1
	-0.5	62.6	13.2	24.2
	-0.7	70.3	16.5	13.2
	-1.5	46.6	9.33	44.1
	-1.9	56.9	10.1	33.0
10	-0.4	62.5	19.6	18.0
	-0.6	73.1	12.3	14.6
	-0.8	67.8	14.3	17.9

Table S8. Quantification of the deconvoluted C 1s spectra (Figure S18a-c, left panels), calculated Mo:$C_{carbidic}$ ratios, and oxide film thicknesses as functions of applied potentials *E* and electrolyte pH values.

Sample		C-species / at. %					Data Evaluation			
pH	*E* / V_{SHE}	Carbide	C-C, C-H	C-OH, C-O-C	C=O	O-C=O	Mo:$C_{carbidic}$ ratio	Interval	oxide film thickness / nm	Interval / nm
3.7	-0.2	15.2	62.8	17.7	2.3	2.1	**2.76**	2.85-2.70	**0.46**	0.52-0.44
	-0.4	14.2	69.6	11.4	3.5	1.3	**2.58**	2.60-2.42	**0.37**	0.39-0.29
	-0.6	19.0	61.1	13.4	4.0	2.5	**2.29**	2.40-2.25	**0.21**	0.27-0.18
	-0.8	5.6	50.3	28.7	12.1	3.3	**2.28**	2.40-2.20	**0.20**	0.27-0.15
7	-0.3	16.4	61.5	16.4	3.2	2.6	**2.64**	2.73-2.56	**0.41**	0.45-0.36
	-0.5	8.8	56.2	24.0	7.5	3.5	**2.40**	2.42-2.32	**0.27**	0.29-0.23
	-0.7	10.2	64.7	20.4	2.1	2.6	**2.31**	2.42-2.25	**0.22**	0.29-0.18
	-1.5	8.0	72.8	10.8	3.8	4.6	**2.36**	2.44-2.32	**0.25**	0.30-0.23
	-1.9	7.7	66.2	16.6	4.4	5.0	**2.29**	2.35-2.25	**0.21**	0.24-0.18
10	-0.4	12.1	52.9	30.0	2.2	2.8	**2.59**	2.70-2.46	**0.38**	0.44-0.31
	-0.6	7.0	72.8	15.9	2.0	2.6	**2.39**	2.45-2.35	**0.27**	0.30-0.24
	-0.8	9.2	57.8	26.8	3.1	3.2	**2.28**	2.40-2.13	**0.20**	0.27-0.10

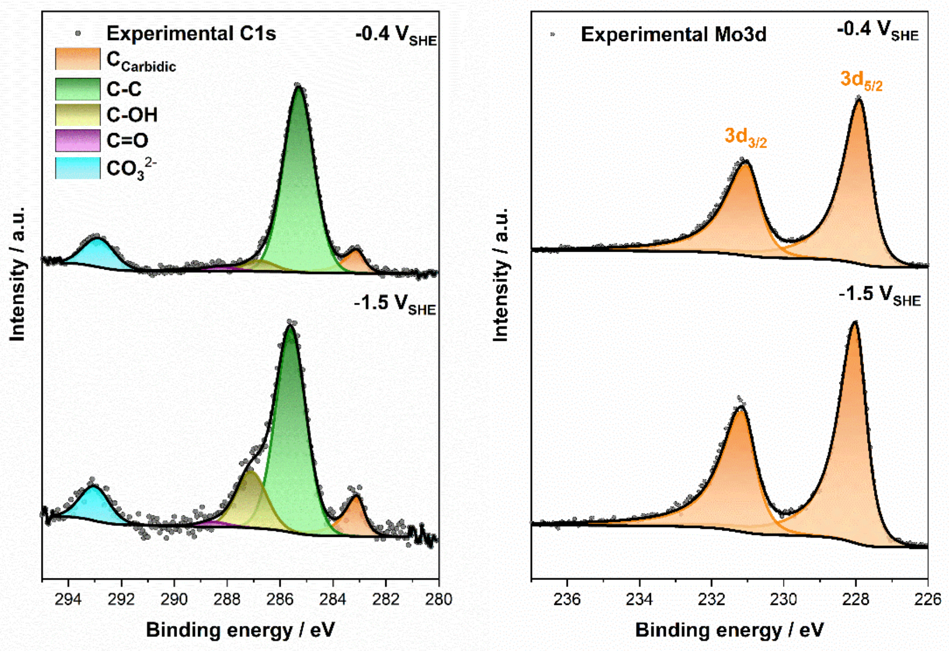

Figure S20. High resolution XPS spectra of Mo_2C after emersion from CO_2 saturated electrolyte. (a) depicts the deconvoluted C1s spectra and (b) shows the corresponding Mo 3d signals. Spectra were acquired at a takeoff angle of 60°.

Table S9. Fit parameters for the deconvolution of the C 1s region. The fits relate to Figure S20. The Mo 3d peak was integrated over the whole region, as a reliable deconvolution was not possible.

Sample		Binding energy [FWHM] / eV						
pH	E / V_{SHE}	Carbide	C-C, C-H	C-OH, C-O-C	C=O	CO_3^{2-}	Mo $3d_{5/2}$	Mo $3d_{3/2}$
6.9	-0.4	283.1 [0.70]	285.3 [1.36]	286.8 [1.36]	288.3 [1.36]	292.9 [1.39]	227.9 [0.75]	231.0 [0.91]
	-1.5	283.1 [0.70]	285.6 [1.36]	287.1 [1.36]	288.6 [1.36]	293.0 [1.30]	228.0 [0.73]	231.2 [0.90]
Constraints	Peakshape	LA(1.1,2.3,2)	GL(30)				LA(1.1,2.3,2)	LA(1.1,2.3,2)
	Asymmetry index	0.3515	0	0	0	0	0.3515	0.3515
	Binding energy	283.1	arbitrary	[C-C] + 1.5	[C-C] + 3	arbitrary	arbitrary	$Mo_{5/2}$ +3.15
	FWHM	0.70-0.85	arbitrary	[C-C] * 1	[C-C] * 1	arbitrary	arbitrary	arbitrary

Table S10. Quantification of the XPS spectra of Mo_2C electrodes recorded at a 60° take-off angle after polarization in CO_2 saturated 0.1 M $KHCO_3$ at pH 6.9 for the determination of the overall atomic fraction.

Sample		Atomic fraction / at.%		
pH	E / V_{SHE}	C	Mo	O
6.9	-0.4	69.2	13.4	17.4
	-1.5	65.2	15.6	19.2

Table S11. Quantification of the deconvoluted C 1s spectra (Figure S20a), calculated Mo:Ccarbidic ratios, and oxide film thicknesses as functions of applied potential after emersion from CO_2 saturated 0.1 M $KHCO_3$.

Sample		C-species / at. %					Data Evaluation			
pH	E / V_{SHE}	Carbide	C-C, C-H	C-OH, C-O-C	C=O	O-C=O	Mo:$C_{carbidic}$ ratio	Interval	oxide film thickness / nm	Interval / nm
6.9	-0.4	7.7	73.5	4.3	1.9	11.9	**2.50**	2.58-2.46	**0.33**	0.38-0.31
	-1.5	10.2	61.23	17.0	1.5	10.0	**2.34**	2.40-2.27	**0.24**	0.27-0.19

Surface pH correction

The pH change in the electrolyte towards the electrode surface is generally described by the Poisson-Nernst-Planck (PNP) equations.[26] In order to estimate the surface pH from steady state current densities recorded in potentiostatic experiments (Figure S21 and Table S12), these equations were solved under simplifying assumptions.

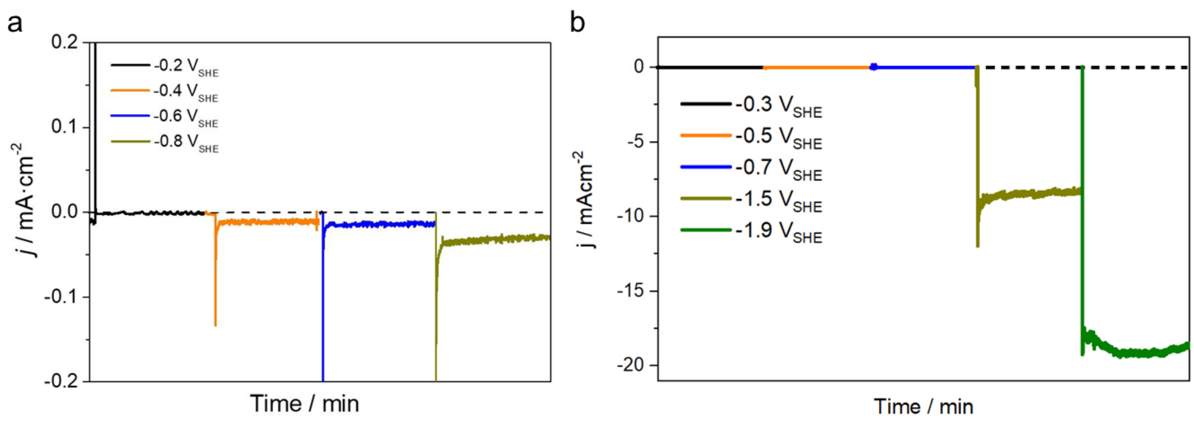

Figure S21. Current transients recorded in hanging meniscus configuration in 0.1 M $NaClO_4$ at (a) pH 3.7 and (b) pH 7 after saturation with Ar at different potentials.

Table S12. Steady state current densities measured in the potentiostatic polarization experiments (see Figure S21) for the calculation of the surface pH correction ΔpH as function of the applied potential and the pH of the bulk electrolytes. The profiles yielding the calculated pH shift are shown in Figure S22.

Sample			
pH	E / V_{SHE}	j / mA·cm^{-2}	surface correction ΔpH
3.7	-0.2	0.000	0
	-0.4	-0.010	+0.13
	-0.6	-0.013	+0.38
	-0.8	-0.045	+2.9
7	-0.3	0.000	0
	-0.5	0.000	0
	-0.7	-0.005	0
	-1.5	-8.3	> +6
	-1.9	~ -18.7	-
10	-0.4	0.000	0
	-0.6	0.000	0
	-0.8	-0.051	+1.9

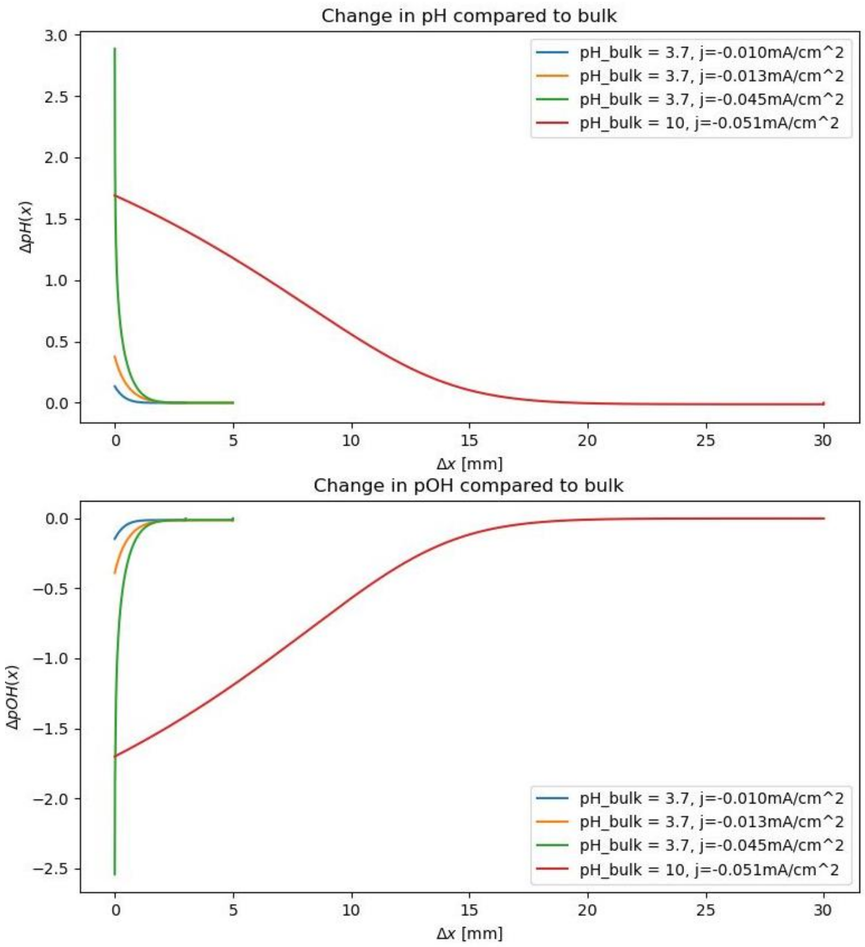

Figure S22. Difference between the surface pH/pOH and the pH/pOH of the bulk electrolyte as functions of the distance to the electrode at acidic and alkaline bulk pH values (3.7 and 10) and electrode current densities j in the potentiostatic experiments (see Table S12).

The supporting $NaClO_4$ electrolyte is assumed to cause the electrode potential to drop within the thin Stern layer at the interface of several nanometers thickness.[27] On the much longer length scales of the interfacial pH change (~1 mm, c.f. Figure S22), the potential is therefore assumed to have dropped instantly and electroneutrality is assumed in solution. Electromigration of the pH-relevant minority species H_3O^+ and OH^- is neglected accordingly, as are all other possible convection effects. Note though that the performed XPS measurements prevent the use of a rotating disc electrode (RDE) usually employed in similar setups to inhibit mass convection in a thin film of controllable thickness in front of the electrode. Nevertheless, there is no indication from experiment that would motivate explicit consideration of convection. The electrode is furthermore assumed to be sufficiently large and flat to model the respective ionic concentrations as one-dimensional functions of the vertical distance away from the electrode.

The HER, constituting the boundary condition at the electrode interface, is described by the reduction of protons $2H_3O^+ + 2e^- \rightarrow H_2$ in the acidic limit and by the reduction of water $2H_2O + 2e^- \rightarrow 2OH^-$ in the alkaline limit. This results in the consumption of one H_3O^+ per electron at low pH values compared to the formation of one OH^- per electron at high pH values. The measured current density is assumed to explicitly relate to the HER.

Under these simplifications, the following system of partial differential equations (PDE) and boundary conditions representing diffusion of the two ionic species H_3O^+ and OH^-can be formulated. This system of equations is solved subject to the boundary conditions at the electrode interface and the bulk limit described in the following.

The low current density acidic and alkaline bulk pH simulations are initialized with a constant profile at the respective ionic bulk concentrations obtained from the pH value and propagated in time until a steady state is reached. The high current density, bulk neutral case is initialized with a profile with increasing pH towards the electrode surface to guarantee numerical stability of the simulation. All simulations are performed employing a finite element solver implemented in Python using the FEniCS computation platform[28] (version 2019.1.0). A mixed function space of linear Lagrange elements is employed and the resulting set of equations is solved by a sparse LU matrix decomposition. Individual parameters can be found in Table S13.

$$\frac{\partial c_{\mathrm{H_3O^+}}}{\partial t} - D_{\mathrm{H_3O^+}} \frac{\partial^2 c_{\mathrm{H_3O^+}}}{\partial x^2} - K_{wb} + K_{wf} c_{\mathrm{H_3O^+}} c_{\mathrm{OH^-}} = 0 \tag{10}$$

$$\frac{\partial c_{\mathrm{OH^-}}}{\partial t} - D_{\mathrm{OH^-}} \frac{\partial^2 c_{\mathrm{OH^-}}}{\partial x^2} - K_{wb} + K_{wf} c_{\mathrm{H_3O^+}} c_{\mathrm{OH^-}} = 0, \qquad \text{in } \Omega \tag{11}$$

with the diffusivity of hydronium $D_{\mathrm{H_3O^+}}$= 9.311 10^{-9} m^2/s and hydroxide $D_{\mathrm{OH^-}}$= 5.273 $10^{-9} m^2/s$ in water at 25°C,[10] the forward K_{wf} = 1.4 10^{11} $(M/s)^{-1}$ and backward K_{wb} = 2.6 10^5 M/s reaction constants for the autoprotolysis of water,[29] *t* and *x* denoting time and space and c_* representing the respective ion concentrations.

The Dirichlet boundary conditions at the bulk limit are given by

$$c_{\mathrm{H_3O^+}} = c^b_{\mathrm{H_3O^+}} \tag{12}$$

$$c_{\mathrm{OH^-}} = c^b_{\mathrm{OH^-}} \quad \text{on } \partial\Omega_D \qquad . \tag{13}$$

Here, c^b_* denotes the respective bulk concentrations related to the bulk pH value according to

$$c^b_{\mathrm{H_3O^+}} = 10^{-pH} \tag{14}$$

$$c^b_{\mathrm{OH^-}} = 10^{-14+pH} \tag{15}$$

The non-linear boundary conditions at the electrode interface are stepped forward in time and implemented as iterative Neumann boundary conditions

$$\frac{\partial^2 c^{t_n}_{\mathrm{H_3O^+}}}{\partial x^2}\Big|_{x=x_0} = \frac{F|j|}{D_{\mathrm{H_3O^+}}} f(c^{t_{n+1}}_{\mathrm{H_3O^+}}) \tag{12}$$

$$\frac{\partial^2 c^{t_n}_{\mathrm{OH^-}}}{\partial x^2}\Big|_{x=x_0} = \frac{F|j|}{D_{\mathrm{OH^-}}} f(c^{t_{n+1}}_{\mathrm{OH^-}}) \qquad , \tag{13}$$

with the switching function *f* (Figure S23) given by a hyperbolic tangent multiplied with a tampered cosine window function regulating the flux of ions at the interface. The index *t* represents the timestep label, *j* the electrode current density and *F* = 96 485.3329 C/mol the Faraday constant. The phenomenological switching has the purpose of continuously connecting the extremal acidic and alkaline reaction pathways and to contain the concentrations and thus the pH value inside the autoprotolytic window between 0 and 14.

Alkaline and acidic bulk pH: Figure S22 depicts the computed surface pH change calculated from the steady state current densities from the potentiostatic experiments (Table S12). Especially at pH 3.7,

and at the most negative potential (-0.8 V_{SHE}) at pH 10, a significant difference between the pH of the bulk electrolyte and at the electrode surface occur. Table S13 furthermore shows the fast convergence into steady state for acidic values compared to the alkaline case.

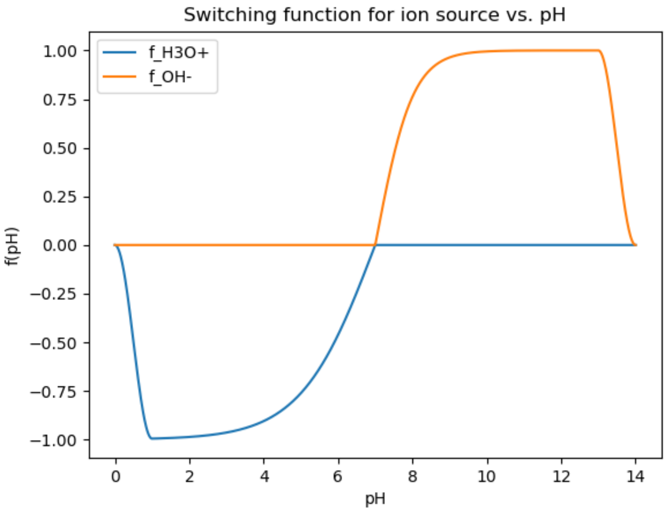

Figure S23. Switching function *f* for ion flux at the interface.

Table S13. Parameters and time to reach a steady state for the diffusion simulations.

pH bulk	j / (mA/cm^2)	x_{bulk} / mm	Δx / µm	Δt / s	T steady state / s
3.7	-0.01	3	5	0.01	19
3.7	-0.013	5	5	0.1	32
3.7	-0.45	5	5	0.0005	60
7	-8.3	7	5	0.5	3600*
10	-0.51	50	5	2	10000

* At the high current for pH=7 no true steady state could be reached.

Figure S24 shows the results of the surface pH calculation for a neutral bulk pH of 7 and an electrode current of $-8.3\ mA/cm^2$.

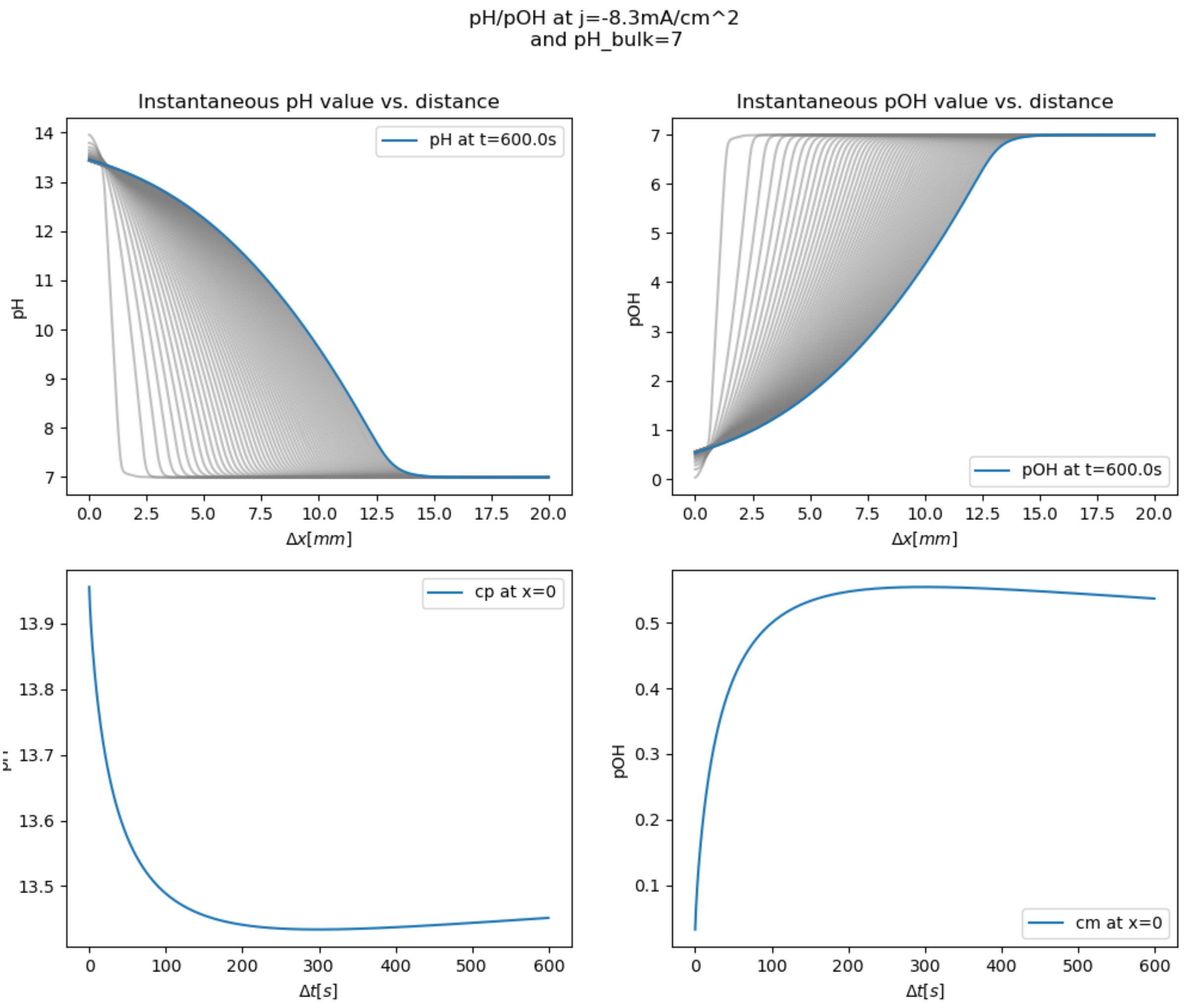

Figure S24. Difference between the surface pH/pOH and the pH/pOH of the bulk electrolyte as functions of the distance to the electrode at the different bulk pH values (of 7) and electrode current densities j in the potentiostatic experiments (see Table S9). The blue curves show the pH evolution after five minutes, each grey line represents a ten second step.

Neutral bulk pH: At a bulk pH of 7, high alkaline pH values between 13-14 are calculated in front of the electrode surface. The results are presented in Figure S24. Within the model described above, the extremely alkaline surface conditions originate from the significant OH^- source at the interface due to a high electrode current of -8.3mA/cm^2. This source cannot be balanced by diffusion any longer as it is the case for the low current boundary conditions considered before. Furthermore, the electrolyte can no longer be treated as an ideal solution in this pH regime and molar concentrations need to be corrected for activities to be directly comparable with the free energy scales in a thermodynamic Pourbaix-Diagramm.

The approximate transport model used here also starts to fail for OH^-concentrations larger than 0.1 mol/l, indicated by our use of the model switching function given in Figure S23, where further chemical processes at the surface as well as correlation in the dynamics of the hydrated ions need to be considered. The latter effect has traditionally been treated within approximate bulk models yielding e.g. the extended Debye Hückel correction or by advanced empirical models like the Pitzer equations.[30]

We note that such corrections exhibit a correct trend compared to the computed Pourbaix diagram already at pH=13 (an extended Debye-Hückel correction predicts an additional interfacial pH increase of about +0.1) but their validity directly at the interface and for extremely high alkaline electrolyte concentrations is questionable.

The surface pH corrections ΔpH resulting from the different simulations are summarized in the last column of Table S12. These corrections have been applied in Figure 2 of the main text to show the effective reaction conditions probed in the quasi in-situ XPS measurements

Supporting Note 5

CO_2 electro-reduction in non-aqueous electrolyte

For the measurements in organic electrolyte, the Mo_2C electrodes were prepared in a home-built quartz tube furnace and transported under H_2 gas atmosphere into the glovebox (MB 200B Eco, MBraun). Potential cycling was carried out inside an Ar-filled glovebox, in a commercial three electrode glass cell in hanging meniscus configuration. As CE served a flame annealed carbon rod (Ultra Carbon Corporation), and an activated carbon quasi reference[31] was used as RE. Electrodes were immersed at a potential of -0.58 $V_{Fc/Fc+}$ and cycled at a scan rate of 20 mV s^{-1} in Ar and CO_2 saturated acetonitrile (99.8 %, anhydrous, Sigma Aldrich) containing 0.1 M tetrabutylammonium hexafluorophosphate ($TBAPF_6$, > 98 %, Molekular). The water content of the acetonitrile was determined by Karl Fischer titration (C20 Coulometric KF Titrator, Mettler Toledo, see Table S11). To determine the potential of the quasi RE in acetonitrile, ferrocene (99.5 %, Alfa Aesar) was added to the solution, and a CV was recorded (Figure S23) in CO_2 saturated electrolyte.

Table S14. Results of the Karl Fischer titration for determination of the water content of acetonitrile.

measurement / #	water content / ppm
1	40.9
2	43.7
3	43.9
**average**	**42.8**
**standard deviation**	**± 1.7**

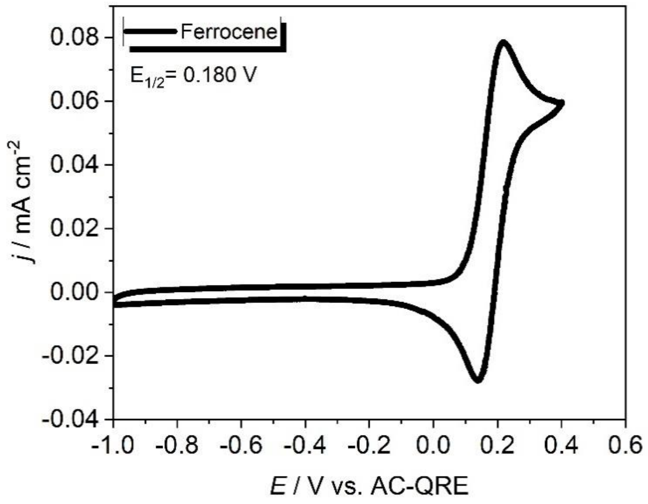

Figure S25. CV of ferrocene/ferrocenium in acetonitrile, where a redox potential of 0.18 V is measured versus the activated carbon quasi reference electrode (AC-QRE) in an Ar-filled glovebox. The CV in Figure 5 in the main manuscript has been calibrated with this value.

For the investigation of potential product formation in organic electrolyte electrochemical IR spectroscopy (EC-IRRAS) with a thin-layer cell configuration was performed in Ar and CO_2 saturated electrolyte (Figure S26) the setup is described in detail in the supporting information of ref. [32].

A Perkin Elmer Spectrum 2000 FT-IR spectrometer equipped with a liquid N_2 cooled mercury cadmium telluride photodetector was used for spectra acquisition. The potential was alternated between the reference potential (E_{ref} = -1.0 $V_{Fc/Fc+}$) and the sample potential (E). Prior to each cathodic polarization step the working electrode was kept at the reference potential for 60 s, followed by acquisition of a reference spectrum. All single beam spectra were recorded by adding 100 scans with a resolution of 1 cm^{-1}. Difference spectra were obtained by subtracting and normalizing ($\Delta R/R = (R(E) - R(E_{ref})) / R_{ref}$) the single beam spectra. Accordingly, in the difference spectra, positive bands correspond to the consumption and negative bands to the formation of species at the sample potential E. All spectra were recorded with p-polarized light, which is sensitive to adsorbed, as well as solution species, therefore a linear polarizer (Edmund optics) was utilized. In the thin-layer cell the same electrolyte and electrodes, as for the measurements in organic electrolyte (see Supporting Note 5) were used.

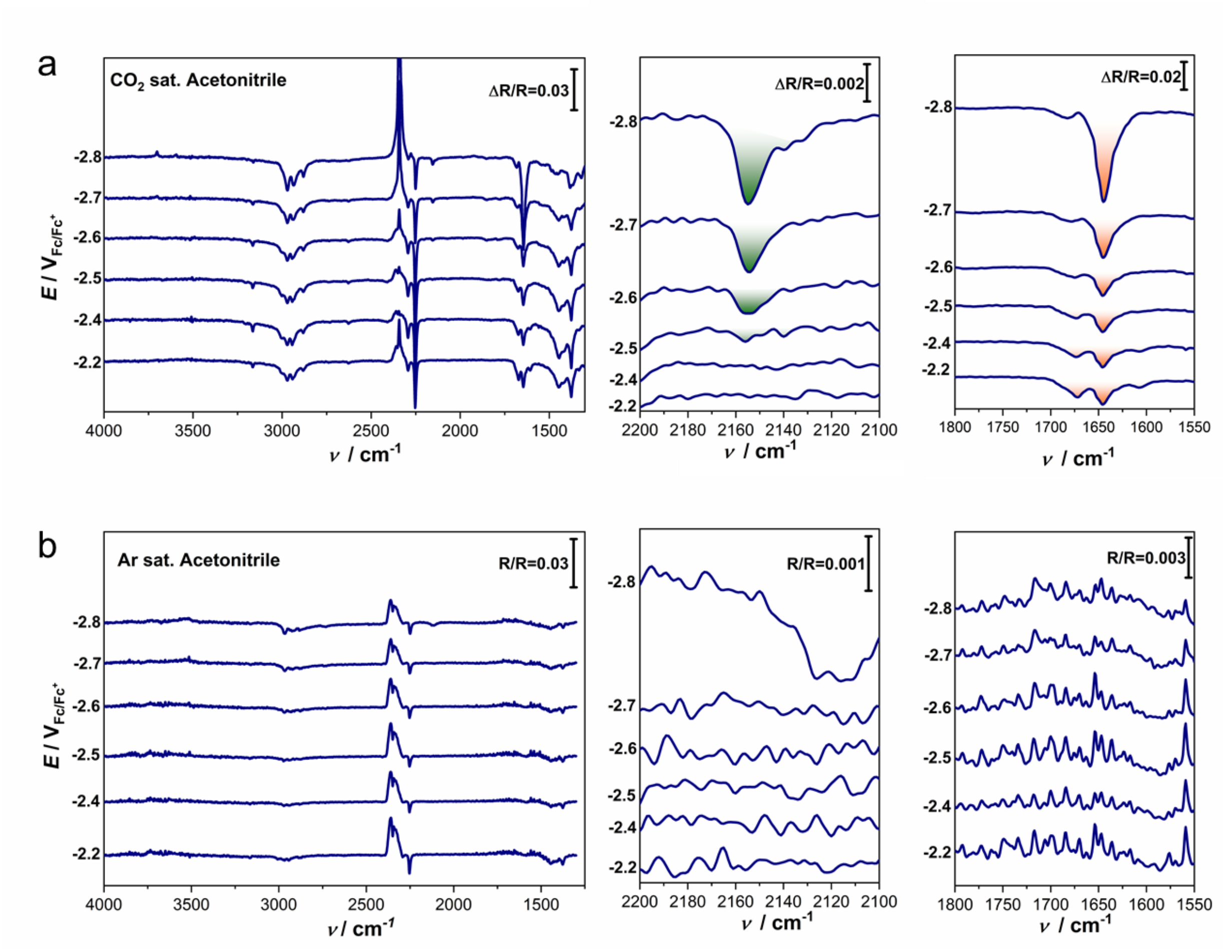

Figure S26. In situ electrochemical infrared reflection absorption spectroscopy (EC-IRRAS) difference spectra recorded in CO_2 (a) and in Ar (b) saturated acetonitrile with 0.1 M tetrabutylammonium hexafluorophosphate ($TBAPF_6$).

Two distinct bands can be assigned to CO_2 reduction product formation, one at ~2158 cm^{-1} (Figure S26a, middle panel) that starts occurring at potentials ≤-2.5$V_{Fc/Fc}$+ (≤ -1.9 V_{SHE}) and can be attributed to the formation of CO.[33] At even more positive potentials, features between 1600 cm^{-1} and 1700 cm^{-1}, associated with the formation of oxalate[33] or carbonate[34], are visible. The exact correlation of these species is foreseen in future work. The formation of products agrees with the depletion of dissolved

CO_2 in the electrolyte, which can be observed as positive band at ~2342 cm^{-1} (asymmetric O-C-O stretching mode) of dissolved CO_2.[35]
The reference spectrum (Figure S26b) shows a signal at ~2250 cm^{-1}, related to acetonitrile,[36] and a double peak at ~2340 cm^{-1} and 2360 cm^{-1}, typical for gaseous CO_2,[37] which is due to residues of air in the optical path of the spectrometer. Clearly, the reaction products observed in the presence of CO_2 are missing in Ar saturated electrolyte.

Supporting Note 6

Synthesis of Mo_2C films

The substrates were cut from polycrystalline Mo rods (99.95 %, Advent Ltd.) with diameters of 5 or 10 mm and were mechanically polished on abrasive papers (CarbiMetTM, Buehler, ITW Test and Measurement GmbH) and with diamond suspensions (6 µm, 3 µm, 1 µm, ESCIL). After each polishing step, the samples were rinsed with deionized (DI) water (Milli-Q, 18.2 MΩ, Millipore, Merck) and sonicated in ethanol. Electropolishing was carried out according to a protocol adapted from Zamin *et al.*[38]. Mo was electropolished for 3 min at 7.0 V versus a carbon felt (SIGRACELL® GFD 4.6 EA, SGL Carbon) CE. The electrolyte consisted of a mixture of phosphoric acid (H_3PO_4, 85 %, Merck), sulfuric acid (H_2SO_4, 95-97 %, Merck) and DI water in a volumetric ratio of 5:1:2 containing 0.01 M Mo trioxide (MoO_3, ≥ 99.5 %, Sigma-Aldrich), that was heated to 65-70 °C. Carburization of the Mo electrodes was carried out following a temperature-programmed reaction (TPR) protocol adapted from Tomás-García *et al.*[39]. Substrates were heated in a tube furnace (HST 12/600, Carbolite) equipped with mass-flow controllers (EL-Flow, Bronkhorst) for the gas feed of CH_4 (> 99.9995 %, Linde) and H_2 (> 99.999 %, Messer). The TPR started with a flushing step at room temperature to remove oxygen from the quartz tube reactor. Then, the substrates were heated at 8 °C/min to a final temperature of 750 °C that was maintained for one hour before cooling down to room temperature again. Meanwhile, gas flows were kept at 25 sccm and 100 sccm for CH_4 and H_2, respectively, yielding a CH_4/H_2 volumetric ratio of 1/4. Once the oven started to cool down, the CH_4 feed was stopped while the H_2 flow was increased to 200 sccm, in order to reduce surface contamination through carbon deposition.[17] After cooling down, no further treatment was applied.

Oxide free Mo_2C electrodes used for measurements in the organic electrolyte (see Figure 3 middle panel and Figure 5 in the main paper) were prepared in a home-built, vacuum-assisted quartz furnace with the same temperature program and flow rates as described above. This reactor, however, allowed for transfer of the specimens into the glovebox in H_2 atmosphere after the synthesis.

References

(1) Necas, D.; Klapetek, P. Gwyddion: An Open-Source Software for SPM Data Analysis. *Centr. Eur. J. Phys.* **2012**, *10*, 181–188.

(2) Yamaura, K.; Huang, Q.; Akaishi, M.; Takayama-Muromachi, E. Superconductivity in the Hexagonal-Layered Molybdenum Carbide. *Phys. Rev. B* **2006**, *74* (18), 184510.

(3) Hong, K. H.; McNally, G. M.; Coduri, M.; Attfield, J. P. Synthesis, Crystal Structure, and Magnetic Properties of $MnFe_3O_5$. *Zeitschrift für Anorg. und Allg. Chemie* **2016**, *642* (23), 1355–1358.

(4) Christensen, A. N.; Kvande, H.; Wahlbeck, P. G.; Näsäkkälä, E. A Neutron Diffraction Investigation on a Crystal of Alpha-Mo2C. *Acta Chem. Scand.* **1977**, *31a*, 509–511.

(5) Fries, R. J.; Kempter, C. P. 195. Dimolybdenum Carbide. *Anal. Chem.* **1960**, *32* (13), 1898–1898.

(6) Sathish, C. I.; Guo, Y.; Wang, X.; Tsujimoto, Y.; Li, J.; Zhang, S.; Matsushita, Y.; Shi, Y.; Tian, H.; Yang, H.; Li, J.; Yamaura, K. Superconducting and Structural Properties of δ-$MoC_{0.681}$ Cubic Molybdenum Carbide Phase. *J. Solid State Chem.* **2012**, *196*, 579–585.

(7) Klinger, M.; Jäger, A.; IUCr. *Crystallographic Tool Box* (*CrysTBox*): Automated Tools for Transmission Electron Microscopists and Crystallographers. *J. Appl. Crystallogr.* **2015**, *48* (6), 2012–2018.

(8) Rankin, D. W. H. *CRC Handbook of Chemistry and Physics*, 89th ed.; Lide, D. R., Ed.; Springer US: Boston, MA, 2009; Vol. 15.

(9) Reuter, K. Ab Initio Thermodynamics and First-Principles Microkinetics for Surface Catalysis. *Catal. Letters* **2016**, *146* (3), 541–563.

(10) Haynes, W. M. *CRC Handbook of Chemistry and Physics*, 91st ed.; Haynes, W. M., Ed.; Taylor & Francis Group, 2010.

(11) Carroll, J. J.; Slupsky, J. D.; Mather, A. E. The Solubility of Carbon Dioxide in Water at Low Pressure. *J. Phys. Chem. Ref. Data* **1991**, *20* (6), 1201–1209.

(12) Crovetto, R. Evaluation of Solubility Data of the System CO2 –H2O from 273 K to the Critical Point of Water. *J. Phys. Chem. Ref. Data* **1991**, *20* (3), 575–589.

(13) Opalka, D.; Scheurer, C.; Reuter, K. Ab Initio Thermodynamics Insight into the Structural Evolution of Working IrO_2 Catalysts in Proton-Exchange Membrane Electrolyzers. ACS Catal. **2019**, *9* (6) 4944–4950.

(14) NIST Standard Reference Database Journal of Physical and Chemical Reference Data Reprints | NIST.

(15) Hansen, H. A.; Rossmeisl, J.; Nørskov, J. K. Surface Pourbaix Diagrams and Oxygen Reduction Activity of Pt, Ag and Ni(111) Surfaces Studied by DFT. *Phys. Chem. Chem. Phys.* **2008**, *10* (25), 3722–3730.

(16) Takeno, N. Atlas of E-pH Diagrams: Intercomparison of Thermodynamic Databases. Geological Survey of Japan Open File Report No.419. In *Research Center for Deep Geological Environments*; 2005; p 219.

(17) Weidman, M. C.; Esposito, D. V.; Hsu, Y.-C.; Chen, J. G. Comparison of Electrochemical Stability of Transition Metal Carbides (WC, W_2C, Mo_2C) over a Wide pH Range. *J. Power Sources* **2012**, *202*, 11–17.

(18) Chen, W. F.; Wang, C. H.; Sasaki, K.; Marinkovic, N.; Xu, W.; Muckerman, J. T.; Zhu, Y.; Adzic, R. R.

Highly Active and Durable Nanostructured Molybdenum Carbide Electrocatalysts for Hydrogen Production. *Energy Environ. Sci.* **2013**, *6* (3), 943–951.

(19) Murugappan, K.; Anderson, E. M.; Teschner, D.; Jones, T. E.; Skorupska, K.; Román-Leshkov, Y. Operando NAP-XPS Unveils Differences in MoO_3 and Mo_2C during Hydrodeoxygenation. *Nat. Catal.* **2018**, *1* (12), 960–967.

(20) Carlson, T. A. Basic Assumptions and Recent Developments in Quantitative XPS. *Surf. Interface Anal.* **1982**, *4* (4), 125–134.

(21) Floquet, N.; Bertrand, O.; Heizmann, J. J. Structural and Morphological Studies of the Growth of MoO_3 Scales during High-Temperature Oxidation of Molybdenum. *Oxid. Met.* **1992**, *37* (3–4), 253–280.

(22) Peña, F. de la; Ostasevicius, T.; Fauske, V. T.; Burdet, P.; Jokubauskas, P.; Nord, M.; Prestat, E.; Sarahan, M.; MacArthur, K. E.; Johnstone, D. N.; Taillon, J.; Caron, J.; Furnival, T.; Eljarrat, A.; Mazzucco, S.; Migunov, V.; Aarholt, T.; Walls, M.; Winkler, F.; Martineau, B.; Donval, G.; Hoglund, E. R.; Alxneit, I.; Hjorth, I.; Zagonel, L. F.; Garmannslund, A.; Gohlke, C.; Iyengar, I.; Chang, H.-W. Hyperspy/Hyperspy v1.4.1. *hyperspy/hyperspy v1.4.1*. 2018.

(23) Foelske-Schmitz, A. X-Ray Photoelectron Spectroscopy in Electrochemistry Research. In *Encyclopedia of Interfacial Chemistry*; Elsevier, 2018; pp 591–606.

(24) Subbaraman, R.; Tripkovic, D.; Chang, K. C.; Strmcnik, D.; Paulikas, A. P.; Hirunsit, P.; Chan, M.; Greeley, J.; Stamenkovic, V.; Markovic, N. M. Trends in Activity for the Water Electrolyser Reactions on 3d M(Ni,Co,Fe,Mn) Hydr(Oxy)Oxide Catalysts. *Nat. Mater.* **2012**, *11* (6), 550–557.

(25) St. Clair, T. P.; Oyama, S. T.; Cox, D. F.; Otani, S.; Ishizawa, Y.; Lo, R.-L.; Fukui, K.; Iwasawa, Y. Surface Characterization of α-Mo_2C (0001). *Surf. Sci.* **1999**, *426* (2), 187–198.

(26) Bazant, M. Z.; Chu, K. T.; Bayly, B. J. Current-Voltage Relations for Electrochemical Thin Films. *SIAM J. Appl. Math.* **2005**, *65* (5), 1463–1484.

(27) Brown, M. A.; Goel, A.; Abbas, Z. Effect of Electrolyte Concentration on the Stern Layer Thickness at a Charged Interface. *Angew. Chemie - Int. Ed.* **2016**, *55* (11), 3790–3794.

(28) Alnæs, M. S.; Blechta, J.; Hake, J.; Johansson, A.; Kehlet, B.; Logg, A.; Richardson, C.; Ring, J.; Rognes, M. E.; Wells, G. N. The FEniCS Project Version 1.5. *Arch. Numer. Softw.* **2015**, *3* (100).

(29) Hammes, G. G. Very Fast Reactions in Solution. *Science* **1966**, *151* (3717), 1507–1511.

(30) Kim, H. T.; Frederick, W. J. Evaluation of Pitzer Ion Interaction Parameters of Aqueous Electrolytes at 25°C. 1. Single Salt Parameters. *J. Chem. Eng. Data* **1988**, *33* (2), 177–184.

(31) Auer, A.; Kunze-Liebhäuser, J. A Universal Quasi-Reference Electrode for in situ EC-STM. *Electrochem. Commun.* **2019**, *98*, 15–18.

(32) Auer, A.; Andersen, M.; Wernig, E.-M.; Hörmann, N. G. ; Buller, N.; Reuter, K.; Kunze-Liebhäuser, J. Self-Activation of Copper Electrodes During CO Electro-Oxidation in Alakline Electrolyte. *Nature Catal.* **2020**, *3*, 797–803.

(33) Desilvestro, J.; Pons, S. The Cathodic Reduction of Carbon Dioxide in Acetonitrile: An Electrochemical and Infrared Spectroelectrochemical Study. *Electroanal. Chem.* **1989**, *267*, 207-220.

(34) Christensen, P.A.; Hamnett, A.; Muir, A.V.G.; Freeman, N.A. CO_2 Reduction at Platinum, Gold and Glassy Carbon Electrodes in Acetonitrile: An In-Situ FTIR Study. *J. Electroanal. Chem.*, **1990**, *288*, 197-215.

(35) Chen, S.; Chen, A. Electrochemical Reduction of Carbon Dioxide on Au Nanoparticles: An in Situ FTIR Study. *J. Phys. Chem. C* **2019**, *123*, 23898−23906.

(36) Figueiredo, M.C.; Ledezma-Yanez, I.; Koper, M.T.M. In Situ Spectroscopic Study of CO_2 Electroreduction at Copper Electrodes in Acetonitrile. *ACS Catal.* **2016**, *6*, 2382–2392.

(37) Marinković, N.S.; Hecht, M.; Loring, J.S.; Fawcett, W.R. A SNIFTIRS Study of the Diffuse Double Layer at Single Crystal Platinum Electrodes in Acetonitrile. *Electrochim. Acta* **1996**, *41*, 641-651.

(38) Zamin, M. On the Electropolishing of Molybdenum. *J. Electrochem. Soc.* **1977**, *124* (10), 1558.

(39) Tomás-García, A. L.; Jensen, J. O.; Bjerrum, N. J.; Li, Q. Hydrogen Evolution Activity and Electrochemical Stability of Selected Transition Metal Carbides in Concentrated Phosphoric Acid. *Electrochim. Acta* **2014**, *137*, 639–646.
